# National trends and disparities in retail food environments in the USA between 1990 and 2014

**DOI:** 10.1017/S1368980023000058

**Published:** 2023-05

**Authors:** Jana A Hirsch, Yuzhe Zhao, Steven Melly, Kari A Moore, Nicolas Berger, James Quinn, Andrew Rundle, Gina S Lovasi

**Affiliations:** 1 Urban Health Collaborative, Dornsife School of Public Health, Drexel University, 3600 Market Street 7th Floor Suite, Philadelphia, PA 19104, USA; 2 Department of Epidemiology & Biostatistics, Dornsife School of Public Health, Drexel University, 3215 Market Street, Philadelphia, PA 19104, USA; 3 Department of Epidemiology and Public Health, Sciensano (Belgian Scientific Institute of Public Health), Ixelles, Belgium; 4 Population Health Innovation Lab, Department of Public Health, Environments and Society, London School of Hygiene and Tropical Medicine, London, UK; 5 Built Environment and Health Research Group, Mailman School of Public Health, Columbia University, New York, USA; 6 Department of Epidemiology, Mailman School of Public Health, Columbia University, New York, USA

**Keywords:** Food environment, Race/ethnicity, Nutrition, Disparities, Environmental justice, Spatial patterning

## Abstract

**Objective:**

To describe national disparities in retail food environments by neighbourhood composition (race/ethnicity and socio-economic status) across time and space.

**Design:**

We examined built food environments (retail outlets) between 1990 and 2014 for census tracts in the contiguous USA (*n* 71 547). We measured retail food environment as counts of all food stores, all unhealthy food sources (including fast food, convenience stores, bakeries and ice cream) and healthy food stores (including supermarkets, fruit and vegetable markets) from National Establishment Time Series business data. Changes in food environment were mapped to display spatial patterns. Multi-level Poisson models, clustered by tract, estimated time trends in counts of food stores with a land area offset and independent variables population density, racial composition (categorised as predominantly one race/ethnicity (>60 %) or mixed), and inflation-adjusted income tertile.

**Setting:**

The contiguous USA between 1990 and 2014.

**Participants:**

All census tracts (*n* 71 547).

**Results:**

All food stores and unhealthy food sources increased, while the subcategory healthy food remained relatively stable. In models adjusting for population density, predominantly non-Hispanic Black, Hispanic, Asian and mixed tracts had significantly more destinations of all food categories than predominantly non-Hispanic White tracts. This disparity increased over time, predominantly driven by larger increases in unhealthy food sources for tracts which were not predominantly non-Hispanic White. Income and food store access were inversely related, although disparities narrowed over time.

**Conclusions:**

Our findings illustrate a national food landscape with both persistent and shifting spatial patterns in the availability of establishments across neighbourhoods with different racial/ethnic and socio-economic compositions.

In the USA, poor diet quality constituted the leading risk factor for mortality^(1)^ with wide and persistent disparities in nutrition and related health outcomes^(2)^. National surveys have identified differences across racial/ethnic groups in meeting fruit and vegetable intake as well as specific dietary components including energy intake, dietary fibre, added sugar and Na^(3)^. Gaps in diet quality also exist between lower-income and higher-income individuals and have worsened over time^(4)^. These trends parallel disparities in risk factors for and rates of chronic diseases including CVD and cancer; during recent decades, disparities between low- and high-socio-economic status groups and between racial/ethnic groups have either not improved or worsened^(5–10)^. Between 1980 and 2014, these differences translated into large and growing inequalities in life expectancy among US counties, with variation explained by differences in socio-economic and race/ethnicity factors or behavioural and metabolic risk factors^(11)^.

Research in recent decades has shed light on non-individual factors that create and reinforce nutrition-related disparities^(12)^. Theory and evidence have pointed to supportive food environments, including those with close, easy and affordable access to healthy food options, as critical for nutrition outcomes^(13,14)^. Similar work has highlighted neighbourhood obstacles to healthy diets, including food deserts (i.e. neighbourhoods with limited access to supermarkets or other sources of healthy and affordable food^(15)^) and food swamps (i.e. areas with high density of establishments selling high-calorie fast food and junk food, relative to healthier options^(16)^). Notably, multiple studies have demonstrated patterning of retail food sources by neighbourhood socio-economic and racial/ethnic composition. Substantial evidence shows increases in fast food or other unhealthy options or absence of healthy stores and supermarkets for areas with lower socio-economic status and higher proportions of non-White residents^(17–22)^. Combined, these point to underlying historic and persistent structural inequalities across neighbourhoods that show up in many dimensions of healthy amenities including retail food access. As a result, policies aimed to address retail food access including financing for fresh food options, shifting items available in smaller stores and increasing transportation options^(23–26)^ target these high-need areas.

Almost no work has analysed trends in retail food environments^(19,27–29)^, with little examination at the national level. A national study across urban areas in New Zealand found a decrease in distance and time to both fast-food outlets and supermarkets, with the biggest decrease in distance for supermarkets seen in the most deprived areas^(28)^. However, these patterns may not translate to other settings, including the USA. A study of fast-food and full-service restaurants across the USA found higher proportions of fast-food restaurants out of total restaurants in low-income and predominantly non-Hispanic Black neighbourhoods^(20)^. Yet, this work was cross-sectional and limited to unhealthy food outlets. We found no national research on temporal trends in the equitable distribution of food outlet resources; previous work on disparities in food access has centred around single or multi-city datasets^(17–20)^.

Therefore, we aim to explore national disparities in retail food environment by neighbourhood composition (racial/ethnic and income). Using a national, longitudinal dataset spanning 25 years (1990–2014), we describe changes to disparities in built food environments (retail outlets) across time and space. We hypothesised that national patterns of food access will match single- and multi-city studies (greater access for neighbourhoods with higher income and non-Hispanic White residents). We further hypothesised that retail food environment disparities would mirror increased disparities in nutrition and health outcomes and would differ across geographic region and urbanicity.

## Methods

### Study sample

We compiled retail environment, demographic and socio-economic data between 1990 and 2014 for all census tracts (‘tracts’) in the contiguous USA (*n* 72 538). Tracts were excluded if they contained no land area (i.e. water tracts) or were non-residential (i.e. < 50 residents), leaving 71 547 tracts. For consistency despite boundary changes, all measures were assigned to 2010 census geographies. Sensitivity analyses examined associations only for tracts within Metropolitan Statistical Areas (MSA), and urban areas classified using Rural Urban Commuting Area (RUCA) codes (1; 1 or 2).

### National food environment data and variables

Longitudinal food environment retail business data came from the National Establishment Time Series (NETS) database, licensed from Walls & Associates (Denver, CO). Detailed methods on the cleaning and classification of the NETS data can be found elsewhere^(30)^. Briefly, NETS represents a census of all US businesses and their industrial classification using Standard Industrial Classification (SIC) codes. It is considered one of the most comprehensive databases of US establishments available, with prior studies leveraging NETS to examine food environment^(27,31)^. From NETS, records were categorised first using SIC codes (online Supplementary Table S1). To fully capture national chain stores, we searched for company or trade names that were on the Nielsen (New York) TDLinx list, Technomics/R&I chain name list, or trade channel matching corresponding categories.

The category ‘All Food Stores’ included any location where food can be purchased for off-premise consumption. ‘Healthy Food Stores’ (HF), a subcategory of All Food Stores, was composed of food stores offering a larger selection of healthy food items. We used stores with consistent food offerings across time and space. HF contained the subcategories ‘All Supermarkets’ and ‘Fruit and Vegetable Markets’ where supermarkets represent stores offering a wide variety of food and household items that typically have a wider selection than traditional grocery stores and were defined using SIC codes, sales amounts, employee counts and chain names. We defined ‘Unhealthy Food Sources’ (UF) as both food stores and restaurants (distinguished from stores as selling ready-to-eat items, including those for on-premise consumption) offering primarily unhealthy food items or limited supplies of healthy foods. This included ‘Convenience Stores’, ‘Fast Food Restaurants’, and ‘Bakeries, Candy, and Ice Cream Stores’.

NETS addresses were geocoded for each year a business was open between 1990 and 2014. We calculated annual counts of food establishments per tract and densities by dividing counts by land area (km^2^ with water bodies, from census TIGER files, removed).

### Sociodemographic data

The Longitudinal Tract Database product (LTDB)^(32)^ was used to estimate population density, percent of population in poverty, unemployed, aged 60+ years, aged 75+ years, with high school or college educations, who own their home, recent immigrant (within 10 years), foreign born, and persons who are not proficient in speaking English.

LTDB data were also used for our exposures of interest: racial/ethnic composition (based on percent within different race/ethnic categories) and inflation-adjusted median household income. For consistency with previous work^(33,34)^, we defined a tracts as predominantly one racial/ethnic group if greater than 60 % of residents were of that particular racial/ethnic group (i.e. predominantly non-Hispanic White; predominantly non-Hispanic Black; predominantly Hispanic; predominantly Asian). Tracts that did not fall into any of these categories were classified as racially mixed areas. We also created longitudinal racial composition metrics defined using categories from previous work^(34)^. We used inflation-adjusted income tertiles across all years (1990, 2000 and 2010) to examine socio-economic disparities (low – less than or equal to $43 135, middle – between $43 135 and less than or equal to $61 883, and high – greater than $61 883).

### Statistical analyses and mapping

We calculated means, standard deviations, and frequencies for food densities and sociodemographic characteristics within each year and over time (1990–2014). We mapped densities of retail food environment variables in each year and for change between 1990 and 2014.

Tract and food environment characteristics were compared across categories of racial/ethnic composition. Because food environment variables are counts (number of stores within each tract), we used generalised Poisson regression to examine associations of racial/ethnic composition and income tertiles with number and trajectory of establishments. The count of each category was modelled as a function of time (in 10-year increments), racial/ethnic composition (time-varying), population density (time-varying z-score using mean and sd of 1990–2010 pooled data) and land area as an offset. Models estimated racial differences at baseline (1990), at 25 years (2014) and in linear time trend across the years (1990–2014). Analyses were clustered by tract to account for repeated measures. Exponentiated coefficients for the time trend (modelled yearly) can be interpreted as mean or expected change in stores per land area over 10 years, adjusted for population density. We did not isolate the effects of racial/ethnic composition from socio-economic composition due to strong correlations between the two; instead, we show similarly constructed models for both racial/ethnic composition and income categories.

We performed all analyses in ArcGIS (10.8) and SAS (9.4). Sensitivity analyses examined associations within restricted geographies (see above).

## Results

### Sample characteristics (1990–2014)

We note trends of increasing population density, older adult population and percent with at least a 4-year college education (Table 1). Tracts became more diverse, with increases in percent foreign born, recent immigrants, not proficient in English and non-White races/ethnicities. This pattern was also noted in racial composition categories; while a majority of tracts were predominantly non-Hispanic White in 1990 (79·5 %), far fewer were predominantly non-Hispanic White by 2010 (63·9 %), with 11 751 (16·4 %) changing from predominantly non-Hispanic White between 1990 and 2010 (online Supplementary Table S2). In all years, tracts that were predominantly non-Hispanic Black, Hispanic, and Asian experienced higher population density, poverty, and unemployment as well as lower educational attainment, household income, and owner-occupied housing (online Supplementary Table S3).

**Table 1:** Characteristics of non-water US census tracts included in sample* (*n* 71 547) between 1990 and 2010

	1990		2000		2010	
	Mean	sd	Mean	sd	Mean	sd
Population density (pop/km^2^)†	1833·5	4300·4	2002·7	4570·7	2029·5	4544·8
Percent aged 60+ years	17·0	8·7	16·7	8·3	19·4	9·1
Percent aged 75+ years	5·3	3·9	6·1	4·4	6·4	4·6
Percent persons with high school degree or less	55·0	18·7	49·0	18·8	43·8	18·2
Percent persons with at least a 4-year college degree	20·0	14·7	23·7	16·9	27·4	18·4
Household income, inflation-adjusted (1000 USD)	56·2	25·0	58·8	26·8	56·5	28·1
Percent poverty	13·3	11·8	12·9	11·0	16·0	12·7
Percent unemployed	6·7	4·8	6·2	5·4	10·0	6·2
Percent owner occupied housing units	65·6	21·9	66·5	22·7	64·6	23·0
Percent foreign born	7·5	11·0	10·4	13·2	12·1	13·7
Percent recent immigrants	3·3	6·2	4·4	6·5	4·5	5·9
Percent speak English not well	2·7	5·8	3·9	6·9	5·1	7·9
Percent non-Hispanic White	76·6	27·6	69·6	29·5	64·1	30·3
Percent non-Hispanic Black	11·5	21·8	13·3	22·6	13·5	22·2
Percent Hispanic	8·7	16·5	11·8	19·1	15·2	21·1
Percent Asian	2·4	5·0	3·7	6·9	4·3	8·3
Racial composition categories‡	%	*n*	%	*n*	%	*n*
Predominantly non-Hispanic White (%)	79·5	56 897	71·2	50 944	63·9	45 729
Predominantly non-Hispanic Black (%)	6·1	4337	6·9	4922	6·9	4902
Predominantly Hispanic (%)	3·3	2331	4·8	3431	6·5	4638
Predominantly Asian (%)	< 0·1	30	0·2	118	0·4	250
Mixed racial composition (%)	11·1	7952	17·0	12 132	22·4	16 028

* Census tracts were excluded if they contained no land area (i.e. were water tracts) or had less than fifty residents.

† Harmonised data for the 1990, 2000 and 2010 population census, published in the Longitudinal Tract Database product (LTDB)^(32)^, were used to estimate population density. LTDB data from the 1990 and 2000 population census and the 2008–2012 American Community Survey were used to estimate all other values.

‡ We defined a tract as predominantly one racial/ethnic group if greater than 60 % of residents were any particular racial/ethnic group. Tracts that did not fall into any of these categories were classified as racially mixed areas.

### Change in retail food environment (1990–2014)

**Fig. 1:**
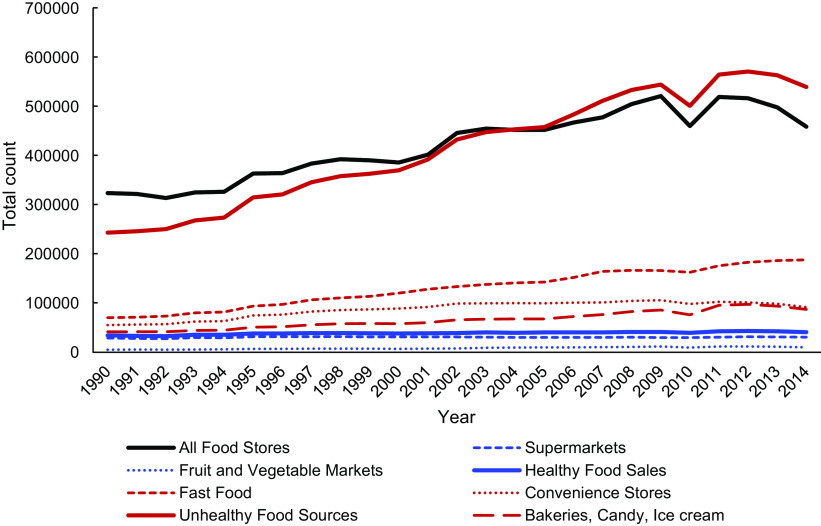
Total count of food retail businesses in the USA by category between 1990 and 2014. *Note: Categories shown are neither mutually exclusive nor collectively exhaustive. For example, All Food Stores includes Unhealthy Food Sources, Supermarkets, Fruit and Vegetable Markets, and Convenience Stores but does not include restaurants. We defined Unhealthy Food Sources as food stores and restaurants (distinguished from stores as selling items primarily ready to eat, including those for on premise consumption), while All Food Stores do not include unhealthy restaurants

The density of All Food Stores increased between 1990 (mean 2·35, sd 7·07 stores/km^2^) and 2014 (mean 3·64, sd 11·41 stores/km^2^) (online Supplementary Table S4). Across all years, there were higher densities of UF than HF (Fig. 1). Notably, UF density more than doubled over the observed time period (mean 1·93 stores/km^2^ in 1990 *v*. mean 4·27 stores/km^2^ in 2014; average change of 2·35 stores/km^2^ between the two times), while HF density remained relatively flat (mean 0·25 stores/km^2^ in 1990 *v*. mean 0·27 stores/km^2^ in 2014; average change of 0·02 stores/km^2^ between the two times). These patterns were retained within most subcategories, with UF (i.e. fast food, convenience stores, bakeries, candy and ice cream) increasing, while supermarkets remained flat or slightly decreased (average change of -0·01 stores/km^2^ between the two time periods). Fruit and vegetable markets were an exception, increasing from an average of 0·06 stores/km^2^ in 1990 to 0·09 stores/km^2^ in 2014 (average increase 0·02 stores/km^2^ between the two times). Examining change by decade, we see slightly larger changes between 2000 and 2010 than 1990 and 2000 (online Supplementary Table S4). Graphing total counts for these retail categories within the USA reveals a steeper rise in UF (red line) compared with the relatively flat HF category (blue line) and a slight dip in all categories around 2009–2011.

**Fig. 2:**
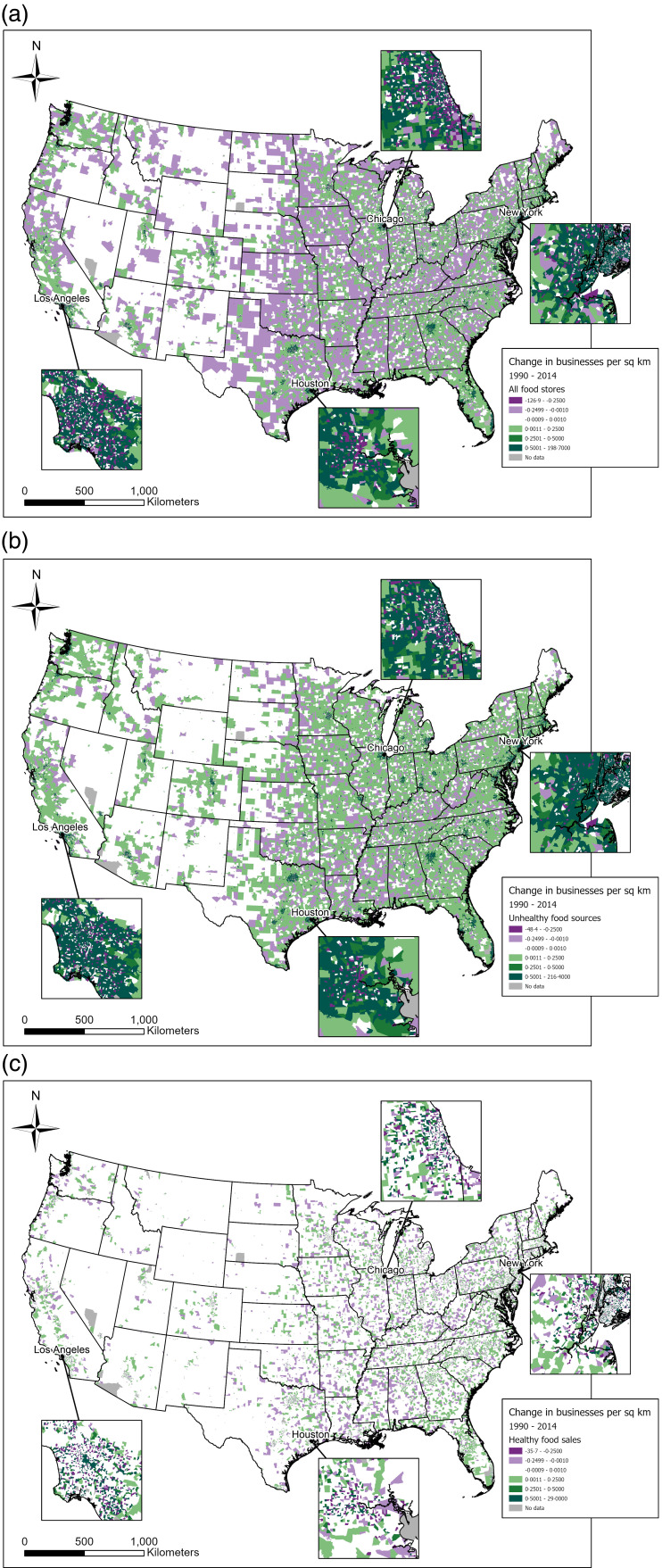
Geographic patterning of change between 1990 and 2014 in density (stores/km^2^) of (a) All Food Stores, (b) Unhealthy Food Sources and (c) Healthy Food Stores. Four largest US cities (in 2010) highlighted (New York, NY; Los Angeles, CA; Chicago, IL; Houston, TX)

When displaying annual densities geographically (not shown), higher densities of all food business categories were generally located in dense population centres. Maps of change between 1990 and 2014 similarly show larger increases in urban areas with most of the larger, rural tracts experiencing relatively little change in either direction (Fig. 2(a)–(c)). As shown in the inlaid maps of the four largest cities, most larger density changes (both increases and decreases) occurred in urban tracts. Additionally, maps illustrate larger increases in unhealthy food (Fig. 2(b) green) compared with the relative stability of healthy food (Fig. 2(c) white). Patterning of change by region of the USA illustrated more stability (lack of change) in the mountain west.

### Retail food environment measures by racial/ethnic composition and socio-economic status

In both 1990 and 2014, tracts that were predominantly non-Hispanic White in 1990 had lower food store density than tracts that were predominantly non-Hispanic Black, Hispanic, Asian or mixed (Table 2). Predominantly non-Hispanic Black, Hispanic, Asian or mixed tracts in 1990 also experienced greater increases in food store density between 1990 and 2014. However, this was primarily driven by UF (increases for all tracts). HF densities declined, on average, between 1990 and 2014 for tracts that were predominantly non-Hispanic Black and predominantly Asian in 1990, with all tracts experiencing declines in supermarket density except predominantly non-Hispanic White tracts (which experienced no change).

**Table 2:** Food environment density measures* over time (1990–2014) by racial composition† in 1990 for non-water US census tracts included in sample‡ (*n* 71 547)

	Predominantly non-Hispanic White		Predominantly non-Hispanic Black		Predominantly Hispanic		Predominantly Asian		Mixed racial composition	
	Mean	sd	Mean	sd	Mean	sd	Mean	sd	Mean	sd
1990										
All food stores	1·56	5·15	4·70	7·87	7·24	12·70	63·17	66·13	5·09	11·28
Healthy food stores	0·17	0·86	0·48	2·02	0·75	2·54	6·48	8·69	0·48	1·74
All supermarkets	0·13	0·61	0·36	1·35	0·52	1·77	3·75	5·18	0·36	1·35
Fruit and vegetable markets	0·04	0·50	0·12	1·20	0·23	1·40	2·74	6·09	0·12	0·88
All unhealthy food sources	1·33	4·52	3·42	5·66	5·41	9·50	45·12	49·88	4·23	9·70
Fast food	0·37	1·30	0·62	1·65	0·86	2·07	6·60	9·07	0·88	2·69
Convenience stores	0·19	0·57	0·48	1·01	0·53	1·23	1·95	3·64	0·49	1·38
Bakeries, candy and ice cream	0·29	1·43	0·36	1·40	0·88	2·35	12·42	14·74	0·75	2·51
2014										
All food stores	2·30	7·48	6·86	14·49	13·28	25·93	92·47	97·55	8·24	18·14
Healthy food stores	0·19	0·82	0·40	1·64	0·94	2·70	5·19	6·84	0·53	1·80
All supermarkets	0·14	0·55	0·26	1·14	0·48	1·75	1·72	3·62	0·34	1·32
Fruit and vegetable markets	0·05	0·51	0·13	0·95	0·46	1·93	3·47	5·18	0·19	1·04
All unhealthy food sources	2·89	9·30	7·40	15·50	13·90	28·69	79·68	78·82	9·39	20·96
Fast food	0·89	2·72	1·85	4·68	3·14	6·79	12·72	13·99	2·36	5·56
Convenience stores	0·34	0·98	0·80	1·56	1·19	2·58	2·45	4·09	0·88	2·08
Bakeries, candy and ice cream	0·52	2·13	0·77	2·52	1·88	4·46	17·58	18·33	1·43	4·34
Change 1990–2014										
All food stores	0·75	3·86	2·16	9·74	6·03	17·23	29·31	50·34	3·15	11·00
Healthy food stores	0·02	0·77	−0·08	1·65	0·18	2·42	−1·30	4·59	0·05	1·66
All supermarkets	0·00	0·55	−0·10	1·31	−0·04	1·60	−2·03	3·91	−0·02	1·31
Fruit and vegetable markets	0·01	0·52	0·01	0·99	0·23	2·14	0·73	3·86	0·06	1·09
All unhealthy food sources	1·56	5·73	3·98	12·36	8·49	22·04	34·57	49·64	5·17	14·17
Fast food	0·52	1·97	1·23	4·38	2·29	6·24	6·11	12·09	1·48	4·50
Convenience stores	0·15	0·88	0·32	1·64	0·66	2·59	0·50	5·29	0·39	2·02
Bakeries, candy and ice cream	0·23	1·60	0·40	2·33	1·00	3·84	5·16	12·16	0·68	3·55


* Food environment metrics derived from National Establishment Time Series (NETS) data 1990–2014 annually. Categories are not mutually exclusive. All Food Stores includes the other store categories (not the retail for consumption on site). Healthy Food Stores includes all Supermarkets and Fruit and Vegetable markets. All Unhealthy Food sources includes Fast Food, Convenience Stores, and the Bakery categories.

† Harmonised data for the 1990 census, 2000 census and the 2008–2012 American Community Survey, published in the Longitudinal Tract Database product (LTDB)^(32)^, were used to estimate all percentage of each race/ethnicity within each tract. We defined a tract as predominantly one racial/ethnic group if greater than 60 % of residents were any particular racial/ethnic group. Tracts that did not fall into any of these categories were classified as racially mixed areas.

‡ Census tracts were excluded if they contained no land area (i.e. were water tracts) or had less than fifty residents.

From multi-level Poisson models, in 1990, predominantly non-Hispanic Black, Hispanic, Asian and mixed racial composition tracts had more food stores than predominantly non-Hispanic White tracts (Table 3). These differences persisted and slightly increased, by 2014 for all tracts aside from predominantly Asian tracts (where the disparity persisted but narrowed). Estimated time trends 1990–2014 showed that almost all tracts, except predominantly Asian, experienced increases in All Food Stores. However, only predominantly non-Hispanic Black, predominantly Hispanic and mixed racial composition tracts experienced increases in HF (1·06 CI 1·01, 1·11; 1·30, CI 1·17, 1·43; and 1·40, CI 1·33, 1·46, respectively). Predominantly Asian tracts experienced a decrease (0·71, CI 0·58, 0·88), and predominantly non-Hispanic White tracts experienced no statistically significant time trend (1·00, CI 0·99, 1·01). All tracts experienced increases in UF, although in predominantly Asian tracts this trend failed to reach statistical significance. When interpreting estimates and CI for predominantly Asian tracts, small sample sizes (*n* 30 in 1990, *n* 118 in 2000, and *n* 250 in 2010) should be considered.

**Table 3:** Racial/ethnic disparities* in food environment measures† over time (1990–2014) for non-water US census tracts included in sample‡ (*n* 71 547)

	Predominantly non-Hispanic White		Predominantly non-Hispanic Black		Predominantly Hispanic		Predominantly Asian		Mixed racial composition	
	Estimate	95 % CI§	Estimate	95 % CI§	Estimate	95 % CI§	Estimate	95 % CI§	Estimate	95 % CI§
Disparities in 1990										
All food stores	Ref		5·69	4·97, 6·52	2·21	1·61, 3·05	110·81	63·12, 194·53	1·28	1·13, 1·45
Healthy food stores	Ref		4·38	3·78, 5·07	1·83	1·32, 2·55	110·70	63·38, 193·37	1·06	0·93, 1·21
All supermarkets	Ref		4·26	3·67, 4·95	1·68	1·21, 2·34	76·15	38·60, 150·22	1·06	0·93, 1·21
Fruit and vegetable markets	Ref		5·00	4·15, 6·03	3·05	2·12, 4·38	292·85	151·78, 565·02	1·12	0·96, 1·31
All unhealthy food sources	Ref		5·58	4·86, 6·41	2·11	1·53, 2·91	87·79	49·07, 157·04	1·36	1·20, 1·55
Fast food	Ref		4·22	3·63, 4·91	1·55	1·12, 2·16	40·89	22·17, 75·42	1·32	1·16, 1·51
Convenience stores	Ref		5·32	4·64, 6·10	1·59	1·15, 2·19	19·04	10·06, 36·05	1·28	1·13, 1·46
Bakeries, candy and ice cream	Ref		2·61	2·23, 3·06	2·10	1·52, 2·91	161·62	85·97, 303·83	1·16	1·02, 1·33
Disparities in 2014										
All food stores	Ref		6·86	5·97, 7·88	3·56	2·77, 4·57	61·81	46·32, 82·49	2·67	2·36, 3·03
Healthy food stores	Ref		4·99	4·30, 5·78	3·43	2·65, 4·45	49·36	34·54, 70·52	2·37	2·08, 2·69
All supermarkets	Ref		4·83	4·16, 5·62	2·20	1·70, 2·84	34·74	23·24, 51·93	2·25	1·98, 2·56
Fruit and vegetable markets	Ref		5·43	4·56, 6·47	7·40	5·59, 9·80	91·81	60·96, 138·26	2·74	2·37, 3·17
All unhealthy food sources	Ref		5·97	5·19, 6·86	2·95	2·29, 3·79	51·46	39·50, 67·03	2·61	2·31, 2·96
Fast food	Ref		5·01	4·33, 5·79	2·45	1·90, 3·16	40·15	30·38, 53·06	2·59	2·28, 2·93
Convenience stores	Ref		7·21	6·29, 8·27	2·55	1·98, 3·28	20·82	15·29, 28·33	2·37	2·10, 2·68
Bakeries, candy and ice cream	Ref		3·92	3·38, 4·53	2·98	2·31, 3·84	62·45	46·49, 83·88	2·44	2·15, 2·76
Time trend 1990–2014										
All food stores	1·13	1·12, 1·13	1·22	1·17, 1·27	1·37	1·25, 1·51	0·88	0·74, 1·06	1·53	1·47, 1·60
Healthy food stores	1·00	0·99, 1·01	1·06	1·01, 1·11	1·30	1·17, 1·43	0·71	0·58, 0·88	1·40	1·33, 1·46
All supermarkets	0·95	0·94, 0·96	1·00	0·95, 1·05	1·06	0·96, 1·17	0·68	0·50, 0·93	1·30	1·24, 1·36
Fruit and vegetable markets	1·23	1·21, 1·25	1·28	1·19, 1·37	1·78	1·58, 2·01	0·76	0·59, 0·98	1·79	1·68, 1·91
All unhealthy food sources	1·34	1·33, 1·35	1·37	1·32, 1·43	1·54	1·40, 1·69	1·07	0·88, 1·30	1·75	1·68, 1·82
Fast food	1·39	1·38, 1·40	1·49	1·42, 1·56	1·68	1·52, 1·85	1·38	1·07, 1·77	1·84	1·76, 1·92
Convenience stores	1·16	1·15, 1·17	1·32	1·27, 1·37	1·42	1·29, 1·55	1·21	0·95, 1·54	1·50	1·44, 1·57
Bakeries, candy and ice cream	1·32	1·31, 1·33	1·56	1·48, 1·64	1·52	1·39, 1·68	0·89	0·71, 1·11	1·79	1·72, 1·87

* Food environment metrics derived from National Establishment Time Series (NETS) data 1990–2014 annually. Categories are not mutually exclusive. All Food Stores includes the other store categories (not the retail for consumption on site). Healthy Food Stores includes all Supermarkets and Fruit and Vegetable Markets. All Unhealthy Food sources includes Fast Food, Convenience Stores and the Bakery categories.

† Harmonised data for the 1990 census, 2000 census and the 2008–2012 American Community Survey, published in the Longitudinal Tract Database product (LTDB), were used to estimate all percentage of each race/ethnicity within each tract. We defined a tract as predominantly one racial/ethnic group if greater than 60 % of residents were any particular racial/ethnic group. Tracts that did not fall into any of these categories were classified as racially mixed areas.

‡ Census tracts were excluded if they contained no land area (i.e. were water tracts) or had less than fifty residents.

§ Estimates derived from generalised Poisson regression models modelled as a function of time (in years), the census tract racial/ethnic composition, the population density and the land area of the tract as an offset.

In 1990, compared with high-income tracts, low-income tracts had approximately 81 % fewer food stores (all types) per land area, controlling for population (0·19, CI 0·16, 0·21) and middle income had 72 % fewer (0·28, CI 0·25, 0·31) (Table 4). Although the gradient remained across all time periods, these disparities narrowed by 2014 (0·45 CI 0·40, 0·51 and 0·33, CI 0·29, 0·38, for low and middle income, respectively). This was driven by increasing time trends for low- and middle-income tracts and a stable time trend for high-income tracts. HF decreased for middle- and high-income tracts but increased for low-income tracts. While all tracts experienced increasing time trends for UF, the increases were greater for low- and middle-income than high-income tracts.

**Table 4:** Income disparities* in food environment measures† over time (1990–2014) for non-water US census tracts included in sample‡ (*n* 71 547)

	Low income		Middle income		High income	
	Estimate	95 % CI§	Estimate	95 % CI§	Estimate	95 % CI§
Disparities in 1990						
All food stores	0·19	0·16, 0·21	0·28	0·25, 0·31	Ref	
Healthy food stores	0·16	0·14, 0·18	0·27	0·24, 0·31	Ref	
All supermarkets	0·17	0·15, 0·19	0·28	0·24, 0·32	Ref	
Fruit and vegetable markets	0·13	0·11, 0·15	0·25	0·22, 0·28	Ref	
All unhealthy food sources	0·17	0·15, 0·19	0·27	0·24, 0·30	Ref	
Fast food	0·15	0·13, 0·17	0·26	0·23, 0·30	Ref	
Convenience stores	0·22	0·20, 0·25	0·34	0·30, 0·39	Ref	
Bakeries, candy, ice cream	0·11	0·10, 0·12	0·21	0·18, 0·23	Ref	
Disparities in 2014						
All food stores	0·45	0·40, 0·51	0·33	0·29, 0·38	Ref	
Healthy food stores	0·44	0·39, 0·51	0·33	0·29, 0·37	Ref	
All supermarkets	0·43	0·38, 0·49	0·32	0·28, 0·36	Ref	
Fruit and vegetable markets	0·51	0·44, 0·59	0·36	0·31, 0·41	Ref	
All unhealthy food sources	0·44	0·39, 0·50	0·33	0·29, 0·37	Ref	
Fast food	0·43	0·38, 0·49	0·33	0·30, 0·38	Ref	
Convenience stores	0·68	0·60, 0·77	0·46	0·40, 0·52	Ref	
Bakeries, candy and ice cream	0·29	0·26, 0·33	0·26	0·23, 0·30	Ref	
Time trend 1990–2014						
All food stores	1·42	1·39, 1·45	1·05	1·02, 1·08	0·97	0·92, 1·03
Healthy food stores	1·29	1·26, 1·32	0·91	0·88, 0·94	0·84	0·79, 0·89
All supermarkets	1·18	1·15, 1·21	0·85	0·82, 0·88	0·80	0·76, 0·85
Fruit and vegetable markets	1·78	1·71, 1·84	1·16	1·11, 1·21	1·00	0·94, 1·06
All unhealthy food sources	1·67	1·63, 1·71	1·23	1·19, 1·26	1·13	1·07, 1·20
Fast food	1·80	1·76, 1·84	1·27	1·23, 1·31	1·15	1·09, 1·22
Convenience stores	1·50	1·47, 1·54	1·07	1·03, 1·10	0·94	0·89, 1·00
Bakeries, candy and ice cream	1·65	1·61, 1·69	1·22	1·18, 1·26	1·10	1·04, 1·17

* Food environment metrics derived from National Establishment Time Series (NETS) data 1990–2014 annually. Categories are not mutually exclusive. All Food Stores includes the other store categories (not the retail for consumption on site). Healthy Food Stores includes all Supermarkets and Fruit and Vegetable Markets. All Unhealthy Food Sources includes Fast Food, Convenience Stores and the Bakery categories.

† Harmonised data for the 1990 census, 2000 census and the 2008–2012 American Community Survey, published in the Longitudinal Tract Database product (LTDB), were used to estimate inflation-adjusted, median household income within each tract. Categories represent tertiles.

‡ Census tracts were excluded if they contained no land area (i.e. were water tracts) or had less than fifty residents.

§ Estimates derived from generalised Poisson regression models modelled as a function of time (in years), the census tract racial/ethnic composition, the population density and the land area of the tract as an offset.

Sensitivity analyses showed similar results for urban areas (measured using MSA or RUCA) but with larger increases over time (online Supplementary Tables S5–S10).

## Discussion

During the period 1990–2014, the prevalence of food stores increased across the USA. Importantly, at every time point, there were higher densities of unhealthy than healthy food retail and steep increases in availability of UF, while HF remained stable. Contrary to expectations, predominantly non-Hispanic Black, Hispanic, Asian and mixed tracts (unadjusted for income) had higher food store densities in 1990 and maintained a denser food environment through 2014 than predominantly non-Hispanic White tracts. In estimated time trends, we see that almost all tracts experienced increases in food stores but that only predominantly non-Hispanic Black, Hispanic and mixed racial composition tracts experienced increases in HF. Across all time periods, low- and medium-income tracts had lower food store density, but the disparity narrowed between 1990 and 2014 (unadjusted for race). Low-income tracts even experienced increases in HF density. Combined, our findings illustrate a national food landscape with both persistent and shifting resources in neighbourhoods with different racial/ethnic and socio-economic compositions.

Reconciling our contrary findings on racial disparities with prior, geographically restricted literature^(21,22,33)^ is challenging. One explanation is a potential conflation of urbanicity and racial/ethnic composition. Specifically, that disparities observed prior in dense, urban settings in the northeast region may not be consistent for patterns across the entire country. This is especially true, since a large portion of census tracts in the USA are rural and predominantly non-Hispanic White. Indeed, our findings match patterns of walkability nationally. In a national study of five walkability indicators in almost 65 000 tracts, neighbourhoods with higher proportion non-Hispanic Black were more walkable (i.e. shorter block length, greater street node density, more developed land use and higher density of street segments)^(35)^. These walkability metrics represent more intensely developed neighbourhoods, which may have higher density of establishments, including all types of food stores. Due to historic and present-day discriminatory housing policies and practices, predominantly non-Hispanic Black, Hispanic, Asian and mixed neighbourhoods exist primarily in intensely developed, urban areas. It is important to consider rural, predominantly non-Hispanic White areas as high need for food access interventions. This rural–urban racial pattern is not isolated to our work; a study of change in fast-food establishments near public schools in California also found differences by urban and rural localities^(19)^. Additionally, results from South Carolina (a largely rural state) show lower minority populations in places with supermarkets and fast-food outlets^(17)^. Interestingly, when we restricted to the year and limited urban geographies of a prior study with similar objectives^(33)^, we find similar patterns of racial/ethnic disparities (less stores for non-White neighbourhoods) found in that paper (not shown). This implies that differences in disparity patterns are not the result of data differences and instead may be time and geographic scale-dependent. Nonetheless, when we restricted analyses to urban areas (defined in multiple ways), the resulting pattern of results was consistent with our national results. This indicates there may be a complicated story across geographies, urbanicity and race. Future work could explore spatial patterns of disparities using geographic weighted regression^(36)^ or stratification by more granular urbanicity and population density measures.

Notably, non-White neighbourhoods had more of both UF and HF. As posited by others, spatial co-occurrence and clustering of establishments may account for some racial/ethnic patterning^(17,37)^. The nuance of results for healthy and unhealthy food establishments shed light on some of the disparities seen previously, as intensely developed spaces (often with more racial/ethnic diversity) had higher UF and larger increases over time. This is consistent with research on ‘food swamps’, fast-food restaurants, or other unhealthy food options^(18,20,37,38)^. In particular, our results align with the national, cross-sectional evidence showing greater proportion fast-food restaurants in low-income and predominantly black neighbourhoods^(20)^. Our results for predominantly Hispanic and Asian tracts suggest that neighbourhoods may be ethnic enclaves with population-specific specialty stores including food stores with culturally appropriate options^(39)^.

The negative gradient we observed between income and food stores is consistent with many cross-sectional studies^(18,20,21,33)^ as well as one, state-level longitudinal analyses of fast-food restaurants^(19)^. Most interestingly, we found a narrowing of income disparities across the 25 years included in this work. This corroborates the national study in New Zealand that found a larger decrease in distance for to supermarkets within over time for the most deprived areas^(28)^. During this period, several national- and local-level policies attempted to address disparities in food environments^(23–26)^. Specifically, the Obama administration (2009–2017) launched the Healthy Food Financing Initiative, modelled after several similar local models^(24,25)^. Notably, in 2011, USDA created their Food Desert Locator (now the Food Access Research Atlas) which combined information on income and access to a supermarket or large grocery store^(15)^. This tool helped governments to channel resources towards increasing healthy food in high-need areas. Our work cannot provide causal evidence around the impact of these policies, and it is unlikely that the number of stores generated by these policies was enough to account for the changes we observed. However, it is encouraging to see the income gap narrowing. Nonetheless, income disparities remain even in the later time periods and UF increased for tracts of all income levels, reinforcing evidence regarding the rise of ‘food swamps’ and increase in unhealthy food options^(17–20,37)^. It is also possible that tertiles did not entirely isolate the lowest resourced tracts where food retail access may have remained unchanged; additional work may benefit from examining income across additional categories (e.g. deciles) to see if patterns remain consistent.

Food retail environment disparities partially parallel worsening gaps in diet quality and chronic diseases. Increasing UF, especially in low-income and predominantly non-Hispanic Black, Hispanic, and mixed neighbourhoods, follow worsened disparities in cardiovascular risk and cancer over recent decades^(5–10)^. Given our findings on healthy food advantage for these neighbourhoods, patterns of diet quality and health may be more driven by access to unhealthy options than access to healthy options^(16,37,38)^. Translating food environment disparities to health highlights other important factors, including transportation, food pricing and cultural acceptability^(40,41)^.

We expand existing literature in several significant ways. To our knowledge, this is the most comprehensive examination of built food environments in the USA; we displayed 25-year patterns for many types of food stores, across the entire contiguous USA. We limited error and bias by leveraging multiple linked databases, years of geoprocessing and comprehensive category refinement. We explicitly demonstrated that disparities in retail food environments exist nationally, rather than within a restricted set of geographies. Further, these patterns may be different than results from smaller areas. We also illustrated ways in which food retail changed longitudinally, including differences across geography, racial composition and income. This demonstrates that disparities in retail food environment are dynamic over time and modifiable. Finally, our multi-level modelling strategy accounted for the outcome variable (counts) and correlations within tracts over time.

Despite this paper’s strengths, research gaps remain. Large spatial and temporal scales required administrative datasets, which may not accurately represent on-the-ground reality^(42)^. Indeed, previous work has shown differences in commercial secondary data sources and stores present during field audits^(43–46)^. Nonetheless, ground-truthing stores historically and nationally is logistically impossible. Future work could utilise crowdsourced data or commercial imagery (i.e. Google Street View) to capture and archive a prospective database of these detailed data^(47)^. Stores were also classified based on assumed purchasing patterns. Yet, we recognise that individuals may purchase healthy foods at locations classified as ‘unhealthy’ (e.g. convenience stores) or purchase unhealthy foods at ‘healthy’ locations (e.g. supermarkets). Importantly, food environments include additional elements beyond retail or business establishments, including marketing, labelling, prices and nutritional quality of the food supply^(48)^. We were unable to account for purchasing patterns, store size, pricing information, or accessibility of culturally appropriate and acceptable foods^(40,49,50)^. As we uncovered here, a complicated story exists for urbanicity, racial composition and food environment subtypes. Additional efforts should leverage advanced modelling techniques, such as latent growth curves^(27)^, to elucidate neighbourhood typologies longitudinally and nationally. Tools such as geographically weighted regression^(36)^ or other advanced spatial methods^(51)^ could identify whether magnitudes of disparities are patterned spatially. Related, racial and ethnic compositions represent a limited view of the complexities of race as a social construct; we were constrained by census-defined categories and by which racial/ethnic categories had large enough populations, densely located, to create ‘predominant’ tracts. Consideration of nuances around intra-group differences and variations in neighbourhood racial composition are important to ensure research encapsulates the complexity of race, ethnicity and racism within the USA. Similarly, median household incomes did not account for household size which may mask differences in purchasing power relevant to food acquisition. Most critically, due to collinearity, we were unable to disentangle racial composition from income distribution. Therefore, all results should be interpreted as the disparity for each component without adjustment for the other. Future work may benefit from the creation of combined categories of income and racial composition (for racial compositions that have enough tracts) to examine potential effect modification. Finally, while we discuss relationships between policies, food environment disparities and nutritional outcome disparities, we caution against discussing causal pathways linking these three outcomes. Future work should explicitly examine these relationships to best understand how policy shifts can impact food environments and subsequent nutrition.

## Conclusion

This research is the first to quantify and display food environment resources nationally across time and to illustrate disparities in change in retail food environments within the USA. Results paint a complicated picture with nuance by racial/ethnic group and type of built food environment measure. Combined, the persistent (and growing) racial/ethnic and socio-economic disparities in unhealthy food access and nutrition-related health outcomes suggest that restricting placement of unhealthy food sources may be more important than support for new, healthy food store locations.

## Supplementary material

For supplementary material accompanying this paper visit https://doi.org/10.1017/S1368980023000058. click here to view supplementary material
