# Supplementary material for: National trends and disparities in retail food environments in the USA between 1990 and 2014
Source: Public Health Nutr. 2023 Jan 16;26(5):1052–62. doi: 10.1017/S1368980023000058 (PMC10191888; doi:10.1017/S1368980023000058)
Supplement: Supplementary file 1 [file S1368980023000058sup001.docx]

# SUPPLEMENTAL TABLES:

| **Table S1: Definitions and Standard Industrial Classification (SIC) codes used to create categories of businesses from the National Establishment Time Series Data (1990-2014) for the Retail Environment and CardioVascular Disease (RECVD) Study** | | | | |
| --- | --- | --- | --- | --- |
| RECVD Name | Category | Definition | Categories Included (if relevant) | SIC Codes or Categories which used Chane Name Searches |
| FSA | All Food Stores | All food stores primarily for off premise consumption. | Wholesale/warehouse – SIC code based definition (WRS)  Wholesale/warehouse – Chain name based definition (WRN)  Convenience Stores – SIC code based definition (CNV)  Convenience Stores – Chain name based definition (CNN)  Convenience Stores/Gas stations – Chain name based definition (CNG)  Gas stations - SIC code based definition (GSS)  Small Grocers/Bodegas (BDS)  Medium size grocers (GRY)  Supermarkets – SIC code based definition (SMK)  Supermarkets – Chain name based definition (SMN)  Supercenters – Chain name based (SCT)  Fish markets (FSH)  Fruit and Vegetable Markets (FVM)  Health food, natural food, vitamin (NAT)  Nuts (NUT)  Meat/dairy (MET)  Bakery, Candy, Ice Cream – SIC code based definition (BKS)  Bakery, Candy, Ice Cream – Chain name based definition (BKN)  Other food stores (OFD)  Conventional Mass Merchandiser – Chain name based definition (CMN)  Discount Department Stores – Chain name based definition (DDP) | Chain name searches (WRN, CNN, CNG, SMN, SCT, BKN, CMN, DDP)  (WRS) 53999906;  (CNV) 54110200-54110299;  (GSS) 55410000, 55419900, 55419901, 55419903, 55419904;  (BDS) 54110000-54110199, 54110300-54119999 AND [employees<5 AND  sales<$2million or sales=missing]. Employees must be non-missing. If employees<5, then sales could be missing. For sales  and employees, the most recent value for these was used if these changed over time.;  (GRY) 54110000-54110199, 54110300-54119999 AND {[employees>=5 AND  employees<25] or employees=missing]] and [sales<$2million or sales=missing]}. If employees is missing and sales is not  missing, then sales must be <$2million. If sales is missing and employees is not missing, then employees is between 5-24. If  both employees and sales is missing, then that record will be coded as ‘GRY’ here. For sales and employees, the most recent  value for these was used if these changed over time.;  (SMK) 54110000-54110199, 54110300-54119999 AND [employees>=25 OR  sales>=$2million]. Sales or employees could be missing as long as at least one of the two is non-missing and meets the  criteria. For sales and employees, the most recent value for these was used if these changed over time;  (FSH) 54210100-54210199;  (FVM) 54310000-54319999;  (NAT) 54990100-54990199;  (NUT) 54419904;  (MET) 54210200-54210299 or 54210000, 54510000, 54519900, 54519901,  54519902, 54519904, 54999902, 54999904;  (BKS) 54610000-54619999 or 54410000, 54419900, 54419901, 54419902,  54419903, 54419905, 54519903, 54999901, 58120200, 58120202, 58120203, 58120204;  (OFD) 54990000, 54990200, 54990202, 54990203, 54990205, 54999900, 54999903, 54999905 |
| HSR | Healthy Food Sales/Stores | Food stores likely offering a larger selection of healthy food items. This contains stores where food is  primarily consumed off premise. | Supermarkets – SIC code based definition (SMK)  Supermarkets – Chain name based definition (SMN)  Supercenters – Chain name based (SCT)  Fruit and Vegetable Markets (FVM) | Chain name searches (SMN, SCT)  (SMK) 54110000-54110199, 54110300-54119999 AND [employees>=25 OR  sales>=$2million]. Sales or employees could be missing as long as at least one of the two is non-missing and meets the  criteria. For sales and employees, the most recent value for these was used if these changed over time;  (FVM) 54310000-54319999 |
| SMA | All Supermarkets | Stores offering a wide variety of food and household items that typically has a wider selection than a  traditional grocery store. A wide variety of fruits, vegetables and low-fat foods are assumed to be available, though unhealthy  food options are also available. Food sold in large markets is typically cheaper than food sold in small grocers. If healthier  food is more expensive than unhealthy food, then supermarkets may encourage healthier food purchases. Includes  supermarkets defined by SIC code + sales + employees OR having a chain name on the TDLinx name list. | Supermarkets – SIC code based definition (SMK)  Supermarkets – Chain name based definition (SMN)  Supercenters – Chain name based (SCT) | Chain name searches (SMN, SCT)  (SMK) 54110000-54110199, 54110300-54119999 AND [employees>=25 OR  sales>=$2million]. Sales or employees could be missing as long as at least one of the two is non-missing and meets the  criteria. For sales and employees, the most recent value for these was used if these changed over time |
| FVM | Fruit and Vegetable Markets | Stand-alone stores that sell primarily fruits and/or vegetables. | Not Applicable | 54310000-54319999 |
| AUR | All Unhealthy Food Sources | Food stores and restaurants offering primarily unhealthy food items or limited supplies of healthy foods. | Convenience Stores – SIC code based definition (CNV)  Convenience Stores – Chain name based definition (CNN)  Convenience Stores/Gas stations – Chain name based definition (CNG)  Bakery, Candy, Ice Cream – SIC code based definition (BKS)  Bakery, Candy, Ice Cream – Chain name based definition (BKN)  Small Grocers/Bodegas (BDS)  Coffee shops – SIC code based definition (CFS)  Coffee shops – Chain name based definition (CFN)  Pizza (PIZ)  Fast Food – SIC code based definition (FFS)  Quick Service Fast food – Chain name based definition (QSV)  Fast Casual Fast food – Chain name based definition (FCS) | Chain name searches (CNN, CNG, BKN, CFN, QSV, FCS)  (CNV) 54110200-54110299;  (BKS) 54610000-54619999 or 54410000, 54419900, 54419901, 54419902,  54419903, 54419905, 54519903, 54999901, 58120200, 58120202, 58120203, 58120204;  (BDS) 54110000-54110199, 54110300-54119999 AND [employees<5 AND  sales<$2million or sales=missing]. Employees must be non-missing. If employees<5, then sales could be missing. For sales  and employees, the most recent value for these was used if these changed over time.;  (CFS) 54990201, 54990204, 58120304;  (PIZ) 58120600, 58120601, 58120602 or SIC Codes in the ranges 58120000-58129999, 54110000-  54999999 that contain “pizza” or “pizzeria”;  (FFS) 58120300-58120303, 58120305-58120399 (all 581203 with the exception of  58130304 – Coffee shops) |
| FFA | Fast Food | Eating places that specialize in low preparation time foods that are eaten cafeteria-style (no waiter service)  or take-away. Fast food is defined by the industry as being "designed for ready availability, use or consumption and sold at  eating establishments for quick availability or take-out. Fast food restaurants are also known as quick-service restaurants. That  definition has low specificity so we added cafeteria style (no waiter service). Foods tend to be highly processed and therefore  high in calories, saturated fat, salt, and sugar. Includes fast food defined by SIC code OR having a chain name on the  Technomics/R&I name list. | Fast Food – SIC code based definition (FFS)  Quick Service Fast food – Chain name based definition (QSV)  Fast Casual Fast food – Chain name based definition (FCS) | Chain name searches (QSV, FCS)  (FFS) 58120300-58120303, 58120305-58120399 (all 581203 with the exception of  58130304 – Coffee shops) |
| CNA | Convenience Stores | National Association of Convenience Stores, an international trade association and publisher of the industry  trade publication Convenience Store News, defines the channel as small stores between 800 and 3,000 square feet, carrying  between 500 and 1,500 SKUs, and meet the following criteria: operating at least 13 hours per day, the store must carry a  limited selection of grocery items including at least two of the following: toilet paper, soap, disposable diapers, pet foods,  breakfast cereal, tuna fish, toothpaste, ketchup, and canned goods. These stores may or may not sell gasoline and offer fast  food services. Assumed to sell mostly highly processed snack food, fast food to go (microwave burgers, etc.) and low quantity  (if any) fresh produce. Includes convenience stores defined by SIC code OR having a chain name on the TDLinx name list. | Convenience Stores – SIC code based definition (CNV)  Convenience Stores – Chain name based definition (CNN)  Convenience Stores/Gas stations – Chain name based definition (CNG) | Chain name searches (CNN, CNG)  (CNV) 54110200-54110299 |
| BKA | Bakeries, candy, ice cream | Generally stand-alone bakeries, candy shops, and ice cream parlors. It is assumed that the majority of these  stores sell high calorie and high sugar foods. Though some of the bakeries may provide fresh breads, it is impossible to  differentiate these stores from providers of cupcakes, cookies, and pastries. This version of bakery/candy/ice cream  enhances the coding that is only defined by SIC by including names of stores that are known chains and may be misclassified  by SIC. Includes bakery, candy, ice cream defined by SIC code OR having a chain name on the Technomics/R&I name list. | Bakery, Candy, Ice Cream – SIC code based definition (BKS)  Bakery, Candy, Ice Cream – Chain name based definition (BKN) | Chain name searches (BKN)  (BKS) 54610000-54619999 or 54410000, 54419900, 54419901, 54419902,  54419903, 54419905, 54519903, 54999901, 58120200, 58120202, 58120203, 58120204 |
| For more information on categories, classification structure, or chain name searches please contact the RECVD team. For information on general methodology see: Hirsch et al 2020 (<https://doi.org/10.1007/s11524-020-00482-2>) | | | | |

| **Table S2: Change in racial composition^a^ between 1990-2010 for non-water US census tracts included in sample^b^ (n=71547).** | | | |
| --- | --- | --- | --- |
| **Change between 1990-2000** | **Frequency** | **Percent** |  |
| All other changes | 2282 | 3.19 |  |
| Changed from predominantly non-Hispanic White | 6220 | 8.69 |  |
| Changed to predominant non-Hispanic White | 267 | 0.37 |  |
| Remained mixed | 5801 | 8.11 |  |
| Remained predominant Asian | 27 | 0.04 |  |
| Remained predominant non-Hispanic Black | 4030 | 5.63 |  |
| Remained predominant Hispanic | 2243 | 3.14 |  |
| Remained predominant non-Hispanic White | 50677 | 70.83 |  |
| **Change between 2000-2010** |  |  |  |
| All other changes | 2946 | 4.12 |  |
| Changed from predominantly non-Hispanic White | 5975 | 8.35 |  |
| Changed to predominant non-Hispanic White | 760 | 1.06 |  |
| Remained mixed | 9281 | 12.97 |  |
| Remained predominant Asian | 108 | 0.15 |  |
| Remained predominant non-Hispanic Black | 4293 | 6 |  |
| Remained predominant Hispanic | 3215 | 4.49 |  |
| Remained predominant non-Hispanic White | 44969 | 62.85 |  |
| **Change between 1990-2010** |  |  |  |
| All other changes | 3644 | 5.09 |  |
| Changed from predominantly non-Hispanic White | 11751 | 16.42 |  |
| Changed to predominant non-Hispanic White | 583 | 0.81 |  |
| Remained mixed | 4676 | 6.54 |  |
| Remained predominant Asian | 22 | 0.03 |  |
| Remained predominant non-Hispanic Black | 3589 | 5.02 |  |
| Remained predominant Hispanic | 2136 | 2.99 |  |
| Remained predominant non-Hispanic White | 45146 | 63.1 |  |
| ^a^ Harmonized data for the 1990 census, 2000 census, and the 2008-2012 American Community Survey, published in the Longitudinal Tract Database product (LTDB), were used to estimate percent of population in each racial/ethnic group. We defined a tract as predominantly one racial/ethnic group if greater than 60% of residents were any particular racial/ethnic group. Tracts that did not fall into any of these categories were classified as racially mixed areas.  ^b^ Census tracts were excluded if they contained no land area (i.e. were water tracts) or had less than 50 residents. | | |  |

| **Table S3: Census tract characteristics^a^ over time (1990-2010) by racial composition^b^ in 1990 for non-water US census tracts included in sample^c^ (n=71547).** | | | | | |
| --- | --- | --- | --- | --- | --- |
| **Racial Composition in 1990** | **Predominantly non-Hispanic White** | **Predominantly non-Hispanic Black** | **Predominantly Hispanic** | **Predominantly Asian** | **Mixed Racial Composition** |
| **1990** | **Mean (SD)** | **Mean (SD)** | **Mean (SD)** | **Mean (SD)** | **Mean (SD)** |
| Population density (pop/km2) | 1174.51 (2827.39) | 4519.39 (6544.36) | 5782.88 (8895.51) | 15762.37 (14601.91) | 3873.78 (6735.87) |
| Percent poverty | 10.07 (8.26) | 31.60 (16.71) | 30.32 (13.38) | 24.39 (10.30) | 21.08 (12.83) |
| Percent unemployed | 5.42 (3.05) | 15.33 (8.14) | 12.68 (5.16) | 8.01 (3.71) | 9.47 (5.49) |
| Percent aged 60+ | 17.80 (9.06) | 15.61 (6.07) | 11.52 (5.49) | 24.35 (8.62) | 13.91 (6.42) |
| Percent aged 75+ | 5.57 (4.12) | 4.70 (2.75) | 3.38 (2.21) | 8.13 (3.99) | 4.47 (3.13) |
| Percent persons with high school degree or less | 52.21 (18.41) | 69.14 (12.65) | 76.31 (11.29) | 68.91 (15.95) | 60.57 (15.92) |
| Percent persons with at least a four-year college degree | 21.90 (15.11) | 10.03 (7.61) | 7.46 (5.90) | 15.71 (9.95) | 15.63 (11.60) |
| Household Income, inflation-adjusted (1,000 USD) | 59.94 (24.98) | 34.87 (18.25) | 37.33 (13.24) | 36.58 (19500.24) | 46.47 (19.63) |
| Percent owner occupied housing units | 70.07 (18.80) | 47.24 (23.17) | 46.67 (24.59) | 19.49 (21.49) | 48.98 (24.20) |
| Percent foreign born | 5.06 (6.14) | 5.32 (9.87) | 35.57 (19.55) | 66.29 (10.31) | 17.99 (15.85) |
| Percent recent immigrants | 1.81 (3.02) | 2.57 (5.09) | 17.78 (12.25) | 37.10 (9.93) | 9.68 (9.99) |
| Percent speak English not well | 1.26 (2.01) | 1.51 (2.53) | 23.80 (11.31) | 40.87 (14.27) | 7.78 (7.41) |
| **2000** |  |  |  |  |  |
| Population density (pop/km2) | 1310.31 (2988.13) | 4342.85 (6503.49) | 6376.75 (9622.23) | 16334.18 (15324.46) | 4344.59 (7399.40) |
| Percent poverty | 9.82 (7.99) | 29.72 (13.86) | 28.43 (11.59) | 25.19 (10.25) | 21.06 (11.54) |
| Percent unemployed | 4.92 (3.86) | 14.53 (7.60) | 12.06 (5.60) | 8.18 (4.42) | 9.47 (6.34) |
| Percent aged 60+ | 17.59 (8.64) | 15.39 (5.59) | 11.45 (5.66) | 25.90 (8.61) | 12.89 (5.98) |
| Percent aged 75+ | 6.49 (4.61) | 5.16 (2.77) | 3.66 (2.42) | 10.24 (4.76) | 4.48 (3.00) |
| Percent persons with high school degree or less | 45.59 (17.95) | 64.34 (12.52) | 73.77 (12.20) | 64.49 (17.40) | 57.81 (16.79) |
| Percent persons with at least a four-year college degree | 26.16 (17.12) | 11.45 (8.25) | 8.82 (7.36) | 20.01 (12.27) | 17.53 (13.76) |
| Household Income, inflation-adjusted (1,000 USD) | 63.15 (26.86) | 35.23 (15.79) | 38.17 (13.20) | 35.25 (19732.14) | 47.08 (19.86) |
| Percent owner occupied housing units | 71.06 (19.76) | 47.38 (22.46) | 47.63 (25.42) | 20.21 (21.84) | 49.64 (24.93) |
| Percent foreign born | 7.62 (8.93) | 7.50 (11.69) | 38.69 (18.13) | 63.98 (12.72) | 23.55 (18.58) |
| Percent recent immigrants | 3.19 (4.70) | 3.09 (4.79) | 15.41 (9.29) | 28.06 (8.46) | 10.57 (9.79) |
| Percent speak English not well | 2.25 (3.51) | 2.37 (3.81) | 24.98 (11.32) | 41.84 (14.81) | 10.63 (9.52) |
| **2010** |  |  |  |  |  |
| Population density (pop/km2) | 1363.18 (3024.22) | 4072.61 (6460.18) | 6307.31 (9447.42) | 15804.36 (13306.35) | 4377.02 (7335.66) |
| Percent poverty | 13.12 (10.36) | 32.49 (15.16) | 29.08 (12.64) | 26.30 (10.66) | 23.61 (13.73) |
| Percent unemployed | 8.84 (4.92) | 19.50 (9.17) | 12.55 (5.97) | 11.04 (5.00) | 12.60 (6.85) |
| Percent aged 60+ | 20.45 (9.23) | 17.27 (6.44) | 13.73 (6.49) | 26.01 (8.62) | 15.02 (7.43) |
| Percent aged 75+ | 6.80 (4.76) | 5.53 (3.22) | 4.37 (3.11) | 11.30 (6.26) | 4.66 (3.71) |
| Percent persons with high school degree or less | 40.64 (17.08) | 56.58 (13.74) | 67.04 (14.52) | 57.96 (18.09) | 52.38 (17.86) |
| Percent persons with at least a four-year college degree | 29.73 (18.46) | 15.26 (11.81) | 12.39 (10.95) | 28.22 (17.47) | 21.34 (16.92) |
| Household Income, inflation-adjusted (1,000 USD) | 60.61 (28.31) | 32.60 (16.85) | 37.19 (14.48) | 39.35 (25867.16) | 45.56 (22.61) |
| Percent owner occupied housing units | 69.22 (20.44) | 45.33 (21.51) | 45.65 (24.80) | 20.93 (21.56) | 48.05 (24.54) |
| Percent foreign born | 9.55 (10.39) | 9.21 (12.67) | 36.69 (17.10) | 59.95 (15.03) | 24.63 (18.35) |
| Percent recent immigrants | 3.58 (4.82) | 3.55 (4.93) | 11.56 (7.88) | 21.59 (10.85) | 9.02 (8.27) |
| Percent speak English not well | 3.31 (4.99) | 3.55 (5.29) | 24.56 (11.84) | 40.70 (15.75) | 12.66 (11.28) |
| ^a^ Harmonized data for the 1990 census, 2000 census, and the 2008-2012 American Community Survey, published in the Longitudinal Tract Database product (LTDB), were used to estimate all variables besides population density (which uses Census 2010).  ^b^ We defined a tract as predominantly one racial/ethnic group if greater than 60% of residents were any particular racial/ethnic group. Tracts that did not fall into any of these categories were classified as racially mixed areas.  ^c^ Census tracts were excluded if they contained no land area (i.e. were water tracts) or had less than 50 residents. | | | | | |

| **Table S4: Density (count/km^2^) of food environment retail establishments between 1990-2014 for non-water US census tracts included in sample^a^ (n=71547).** | | | | | | | | |
| --- | --- | --- | --- | --- | --- | --- | --- | --- |
|  | All Food Stores | Healthy Food Stores | Supermarkets | Fruit and Vegetable Markets | Unhealthy Food Sources | Fast Food | Convenience Stores | Bakeries, Candy, Ice cream |
| **Year** | **Mean (SD)** | **Mean (SD)** | **Mean (SD)** | **Mean (SD)** | **Mean (SD)** | **Mean (SD)** | **Mean (SD)** | **Mean (SD)** |
| 1990 | 2.35 (7.07) | 0.25 (1.20) | 0.19 (0.86) | 0.06 (0.67) | 1.93 (5.90) | 0.46 (1.59) | 0.25 (0.77) | 0.37 (1.67) |
| 1991 | 2.35 (7.10) | 0.24 (1.20) | 0.18 (0.86) | 0.06 (0.67) | 1.96 (5.98) | 0.47 (1.61) | 0.26 (0.79) | 0.37 (1.66) |
| 1992 | 2.38 (7.48) | 0.24 (1.21) | 0.18 (0.86) | 0.06 (0.67) | 2.07 (6.44) | 0.49 (1.68) | 0.27 (0.81) | 0.38 (1.75) |
| 1993 | 2.64 (8.77) | 0.27 (1.37) | 0.20 (0.92) | 0.08 (0.79) | 2.33 (7.55) | 0.53 (1.79) | 0.29 (0.87) | 0.43 (1.98) |
| 1994 | 2.66 (8.80) | 0.27 (1.36) | 0.19 (0.90) | 0.08 (0.80) | 2.37 (7.68) | 0.54 (1.82) | 0.30 (0.88) | 0.43 (2.00) |
| 1995 | 2.97 (9.83) | 0.29 (1.47) | 0.20 (0.93) | 0.09 (0.89) | 2.70 (8.62) | 0.61 (1.96) | 0.34 (0.95) | 0.49 (2.21) |
| 1996 | 2.93 (9.56) | 0.29 (1.46) | 0.20 (0.95) | 0.09 (0.86) | 2.69 (8.47) | 0.62 (1.99) | 0.35 (0.95) | 0.49 (2.21) |
| 1997 | 3.01 (9.58) | 0.28 (1.39) | 0.20 (0.92) | 0.09 (0.83) | 2.81 (8.57) | 0.66 (2.04) | 0.37 (0.96) | 0.51 (2.27) |
| 1998 | 3.03 (9.51) | 0.28 (1.37) | 0.20 (0.92) | 0.08 (0.81) | 2.85 (8.60) | 0.68 (2.12) | 0.38 (0.97) | 0.52 (2.27) |
| 1999 | 2.96 (9.25) | 0.27 (1.33) | 0.19 (0.89) | 0.08 (0.78) | 2.82 (8.42) | 0.69 (2.14) | 0.38 (0.98) | 0.51 (2.21) |
| 2000 | 2.86 (8.82) | 0.27 (1.29) | 0.19 (0.88) | 0.08 (0.75) | 2.81 (8.30) | 0.73 (2.23) | 0.38 (0.98) | 0.50 (2.14) |
| 2001 | 3.00 (9.38) | 0.27 (1.31) | 0.19 (0.87) | 0.08 (0.77) | 2.97 (8.85) | 0.76 (2.28) | 0.40 (1.03) | 0.52 (2.26) |
| 2002 | 3.49 (11.29) | 0.28 (1.38) | 0.19 (0.87) | 0.09 (0.86) | 3.42 (10.58) | 0.79 (2.34) | 0.45 (1.17) | 0.57 (2.39) |
| 2003 | 3.71 (12.25) | 0.29 (1.45) | 0.19 (0.87) | 0.11 (0.93) | 3.65 (11.53) | 0.82 (2.40) | 0.46 (1.23) | 0.59 (2.50) |
| 2004 | 3.69 (12.17) | 0.29 (1.43) | 0.18 (0.84) | 0.11 (0.93) | 3.69 (11.63) | 0.84 (2.45) | 0.46 (1.24) | 0.59 (2.50) |
| 2005 | 3.67 (11.98) | 0.29 (1.42) | 0.18 (0.84) | 0.11 (0.93) | 3.71 (11.55) | 0.85 (2.49) | 0.46 (1.24) | 0.59 (2.45) |
| 2006 | 3.86 (12.84) | 0.29 (1.39) | 0.18 (0.83) | 0.11 (0.92) | 3.95 (12.54) | 0.91 (2.65) | 0.48 (1.30) | 0.63 (2.63) |
| 2007 | 3.97 (13.27) | 0.29 (1.39) | 0.18 (0.83) | 0.11 (0.92) | 4.20 (13.40) | 1.00 (2.91) | 0.48 (1.31) | 0.66 (2.70) |
| 2008 | 4.22 (14.13) | 0.29 (1.40) | 0.18 (0.83) | 0.11 (0.93) | 4.39 (13.98) | 1.00 (2.86) | 0.50 (1.37) | 0.71 (2.89) |
| 2009 | 4.34 (14.44) | 0.29 (1.43) | 0.18 (0.83) | 0.12 (0.98) | 4.46 (14.12) | 0.99 (2.78) | 0.52 (1.42) | 0.73 (2.90) |
| 2010 | 3.70 (12.01) | 0.27 (1.27) | 0.18 (0.80) | 0.09 (0.83) | 3.96 (12.20) | 0.96 (2.71) | 0.47 (1.28) | 0.63 (2.55) |
| 2011 | 4.24 (13.74) | 0.29 (1.31) | 0.18 (0.82) | 0.11 (0.87) | 4.56 (14.28) | 1.07 (3.07) | 0.51 (1.41) | 0.77 (2.94) |
| 2012 | 4.16 (13.18) | 0.29 (1.28) | 0.19 (0.82) | 0.10 (0.83) | 4.58 (14.21) | 1.14 (3.37) | 0.51 (1.42) | 0.77 (2.93) |
| 2013 | 3.93 (12.21) | 0.28 (1.22) | 0.18 (0.80) | 0.10 (0.78) | 4.45 (13.61) | 1.17 (3.49) | 0.49 (1.36) | 0.73 (2.82) |
| 2014 | 3.64 (11.41) | 0.27 (1.16) | 0.18 (0.79) | 0.09 (0.73) | 4.27 (13.11) | 1.19 (3.56) | 0.46 (1.29) | 0.69 (2.67) |
| **Average Change** | | | | | | | | |
| 1990-2000 | 0.51 (3.87) | 0.02 (0.91) | 0.00 (0.67) | 0.02 (0.62) | 0.88 (3.88) | 0.27 (1.38) | 0.13 (0.81) | 0.13 (1.32) |
| 2000-2010 | 0.85 (5.31) | 0.00 (0.91) | -0.01 (0.62) | 0.02 (0.69) | 1.16 (5.51) | 0.24 (1.61) | 0.09 (0.98) | 0.14 (1.53) |
| 2010-2014 | -0.07 (3.15) | 0.00 (0.65) | 0.00 (0.42) | -0.01 (0.49) | 0.31 (3.22) | 0.23 (1.64) | -0.01 (0.80) | 0.05 (1.29) |
| 1990-2014 | 1.29 (6.59) | 0.02 (1.07) | -0.01 (0.79) | 0.03 (0.75) | 2.35 (8.82) | 0.73 (2.84) | 0.21 (1.22) | 0.32 (2.09) |

| **Table S5: Racial/ethnic disparities^b^ in food environment measures^a^ over time (1990-2014) for non-water US census tracts included in urban (defined by MSA) sample^c^ .** | | | | | |
| --- | --- | --- | --- | --- | --- |
|  | **Predominantly non-Hispanic White** | **Predominantly non-Hispanic Black** | **Predominantly Hispanic** | **Predominantly Asian** | **Mixed Racial Composition** |
| **Disparities in 1990** | **Estimate (95% CI)^d^** | **Estimate (95% CI) ^d^** | **Estimate (95% CI) ^d^** | **Estimate (95% CI) ^d^** | **Estimate (95% CI) ^d^** |
| All Food Stores | Ref | 6.39 (5.33, 7.65) | 2.43 (1.50, 3.93) | 53.20 (30.02, 94.27) | 1.44 (1.20, 1.73) |
| Healthy Food Sales | Ref | 4.81 (3.98, 5.82) | 2.00 (1.22, 3.27) | 52.33 (29.71, 92.18) | 1.17 (0.97, 1.41) |
| All Supermarkets | Ref | 4.69 (3.87, 5.68) | 1.87 (1.14, 3.06) | 36.40 (18.49, 71.65) | 1.17 (0.97, 1.41) |
| Fruit and Vegetable Markets | Ref | 5.49 (4.39, 6.85) | 3.10 (1.86, 5.16) | 131.05 (66.51, 258.23) | 1.24 (1.01, 1.51) |
| All Unhealthy Food Sources | Ref | 6.03 (5.02, 7.23) | 2.20 (1.36, 3.57) | 39.90 (22.09, 72.06) | 1.51 (1.26, 1.81) |
| Fast Food | Ref | 4.51 (3.73, 5.46) | 1.56 (0.96, 2.53) | 17.71 (9.58, 32.73) | 1.44 (1.19, 1.74) |
| Convenience Stores | Ref | 5.83 (4.86, 7.00) | 1.72 (1.06, 2.79) | 9.20 (4.83, 17.52) | 1.39 (1.16, 1.67) |
| Bakeries, candy, ice cream | Ref | 2.76 (2.27, 3.36) | 2.08 (1.28, 3.37) | 69.21 (36.54, 131.08) | 1.28 (1.06, 1.53) |
| **Disparities in 2014** |  |  |  |  |  |
| All Food Stores | Ref | 7.06 (5.90, 8.45) | 3.83 (2.73, 5.39) | 25.02 (18.64, 33.56) | 2.56 (2.10, 3.13) |
| Healthy Food Sales | Ref | 5.04 (4.18, 6.08) | 3.63 (2.57, 5.13) | 20.05 (13.95, 28.82) | 2.24 (1.83, 2.74) |
| All Supermarkets | Ref | 4.84 (4.00, 5.86) | 2.36 (1.67, 3.33) | 14.12 (9.39, 21.24) | 2.11 (1.72, 2.58) |
| Fruit and Vegetable Markets | Ref | 5.65 (4.58, 6.96) | 7.71 (5.39, 11.03) | 37.37 (24.69, 56.56) | 2.65 (2.14, 3.28) |
| All Unhealthy Food Sources | Ref | 6.00 (5.01, 7.18) | 3.11 (2.21, 4.37) | 20.36 (15.56, 26.64) | 2.46 (2.02, 2.99) |
| Fast Food | Ref | 4.94 (4.12, 5.94) | 2.55 (1.81, 3.59) | 15.74 (11.88, 20.86) | 2.40 (1.97, 2.93) |
| Convenience Stores | Ref | 7.53 (6.30, 8.99) | 2.88 (2.05, 4.04) | 9.11 (6.67, 12.46) | 2.31 (1.90, 2.82) |
| Bakeries, candy, ice cream | Ref | 3.84 (3.20, 4.61) | 3.01 (2.14, 4.24) | 23.55 (17.46, 31.77) | 2.23 (1.83, 2.72) |
| **Time Trend 1990-2014** |  |  |  |  |  |
| All Food Stores | 1.17 (1.16, 1.19) | 1.23 (1.15, 1.30) | 1.42 (1.30, 1.55) | 0.86 (0.72, 1.03) | 1.49 (1.41, 1.58) |
| Healthy Food Sales | 1.03 (1.02, 1.05) | 1.05 (0.98, 1.12) | 1.32 (1.20, 1.46) | 0.69 (0.56, 0.85) | 1.35 (1.27, 1.44) |
| All Supermarkets | 0.98 (0.97, 1.00) | 1.00 (0.93, 1.07) | 1.09 (0.98, 1.20) | 0.66 (0.49, 0.90) | 1.26 (1.18, 1.34) |
| Fruit and Vegetable Markets | 1.24 (1.22, 1.27) | 1.26 (1.15, 1.37) | 1.81 (1.62, 2.04) | 0.74 (0.57, 0.95) | 1.70 (1.58, 1.84) |
| All Unhealthy Food Sources | 1.38 (1.36, 1.39) | 1.37 (1.29, 1.46) | 1.59 (1.45, 1.73) | 1.04 (0.85, 1.27) | 1.69 (1.59, 1.78) |
| Fast Food | 1.41 (1.39, 1.43) | 1.47 (1.37, 1.56) | 1.73 (1.58, 1.89) | 1.34 (1.05, 1.72) | 1.75 (1.65, 1.85) |
| Convenience Stores | 1.18 (1.17, 1.20) | 1.32 (1.24, 1.40) | 1.47 (1.34, 1.61) | 1.18 (0.92, 1.50) | 1.46 (1.38, 1.55) |
| Bakeries, candy, ice cream | 1.35 (1.33, 1.36) | 1.54 (1.44, 1.65) | 1.57 (1.44, 1.72) | 0.86 (0.69, 1.07) | 1.70 (1.60, 1.80) |
| ^a^ Harmonized data for the 1990 census, 2000 census, and the 2008-2012 American Community Survey, published in the Longitudinal Tract Database product (LTDB), were used to estimate all percentage of each race/ethnicity within each tract. We defined a tract as predominantly one racial/ethnic group if greater than 60% of residents were any particular racial/ethnic group. Tracts that did not fall into any of these categories were classified as racially mixed areas.  ^b^ Food environment metrics derived from National Establishment Time Series (NETS) data 1990-2014 annually.  ^c^ Census tracts were excluded if they contained no land area (i.e. were water tracts) or had less than 50 residents. Urban tracts were defined as falling within an MSA  ^d^ Estimates derived from generalized poisson regression models modeled as a function of time (in years), the census tract racial/ethnic composition, the population density, and the land area of the tract as an offset. | | | | | |
| **Table S6: Racial/ethnic disparities^b^ in food environment measures^a^ over time (1990-2014) for non-water US census tracts included in urban (defined by RUCA=1) sample^c^** | | | | | |
|  | **Predominantly non-Hispanic White** | **Predominantly non-Hispanic Black** | **Predominantly Hispanic** | **Predominantly Asian** | **Mixed Racial Composition** |
| **Disparities in 1990** | **Estimate (95% CI)^d^** | **Estimate (95% CI) ^d^** | **Estimate (95% CI) ^d^** | **Estimate (95% CI) ^d^** | **Estimate (95% CI) ^d^** |
| All Food Stores | Ref | 4.50 (4.12, 4.92) | 2.79 (2.05, 3.81) | 16.53 (9.07, 30.13) | 2.03 (1.74, 2.36) |
| Healthy Food Sales | Ref | 3.33 (2.99, 3.70) | 2.25 (1.63, 3.11) | 16.03 (8.87, 28.98) | 1.60 (1.37, 1.87) |
| All Supermarkets | Ref | 3.22 (2.89, 3.59) | 2.08 (1.49, 2.88) | 11.07 (5.58, 21.97) | 1.58 (1.35, 1.85) |
| Fruit and Vegetable Markets | Ref | 3.96 (3.37, 4.64) | 3.70 (2.60, 5.27) | 42.10 (20.48, 86.55) | 1.77 (1.48, 2.11) |
| All Unhealthy Food Sources | Ref | 4.08 (3.73, 4.46) | 2.44 (1.79, 3.32) | 11.83 (6.37, 21.97) | 2.08 (1.79, 2.42) |
| Fast Food | Ref | 2.92 (2.64, 3.23) | 1.65 (1.20, 2.25) | 4.93 (2.62, 9.26) | 1.93 (1.65, 2.25) |
| Convenience Stores | Ref | 4.21 (3.85, 4.61) | 1.99 (1.46, 2.73) | 2.92 (1.50, 5.69) | 1.96 (1.68, 2.28) |
| Bakeries, candy, ice cream | Ref | 1.79 (1.60, 2.00) | 2.20 (1.61, 3.01) | 19.47 (10.03, 37.79) | 1.70 (1.46, 1.99) |
| **Disparities in 2014** |  |  |  |  |  |
| All Food Stores | Ref | 3.42 (3.11, 3.77) | 3.37 (2.72, 4.16) | 6.49 (4.80, 8.76) | 2.48 (2.26, 2.73) |
| Healthy Food Sales | Ref | 2.41 (2.16, 2.70) | 3.14 (2.52, 3.91) | 5.19 (3.58, 7.51) | 2.13 (1.93, 2.36) |
| All Supermarkets | Ref | 2.25 (2.01, 2.52) | 1.98 (1.59, 2.47) | 3.54 (2.34, 5.38) | 1.96 (1.77, 2.17) |
| Fruit and Vegetable Markets | Ref | 2.98 (2.57, 3.46) | 7.30 (5.73, 9.30) | 10.68 (7.00, 16.27) | 2.75 (2.41, 3.14) |
| All Unhealthy Food Sources | Ref | 2.84 (2.57, 3.13) | 2.66 (2.15, 3.30) | 5.16 (3.92, 6.78) | 2.32 (2.11, 2.56) |
| Fast Food | Ref | 2.27 (2.04, 2.51) | 2.13 (1.71, 2.65) | 3.88 (2.92, 5.16) | 2.20 (2.00, 2.43) |
| Convenience Stores | Ref | 3.83 (3.48, 4.23) | 2.68 (2.16, 3.32) | 2.53 (1.84, 3.48) | 2.34 (2.12, 2.57) |
| Bakeries, candy, ice cream | Ref | 1.77 (1.59, 1.97) | 2.51 (2.02, 3.12) | 5.78 (4.26, 7.83) | 2.07 (1.88, 2.28) |
| **Time Trend 1990-2014** |  |  |  |  |  |
| All Food Stores | 1.21 (1.20, 1.22) | 1.08 (1.04, 1.12) | 1.31 (1.21, 1.42) | 0.82 (0.68, 0.99) | 1.32 (1.26, 1.39) |
| Healthy Food Sales | 1.06 (1.04, 1.07) | 0.92 (0.88, 0.97) | 1.21 (1.11, 1.33) | 0.66 (0.53, 0.82) | 1.19 (1.13, 1.26) |
| All Supermarkets | 1.02 (1.00, 1.03) | 0.88 (0.84, 0.92) | 1.00 (0.91, 1.09) | 0.63 (0.47, 0.85) | 1.11 (1.05, 1.17) |
| Fruit and Vegetable Markets | 1.24 (1.22, 1.27) | 1.10 (1.03, 1.19) | 1.65 (1.47, 1.85) | 0.70 (0.53, 0.92) | 1.49 (1.39, 1.61) |
| All Unhealthy Food Sources | 1.41 (1.40, 1.42) | 1.21 (1.17, 1.26) | 1.46 (1.35, 1.58) | 1.00 (0.81, 1.22) | 1.48 (1.41, 1.55) |
| Fast Food | 1.43 (1.42, 1.44) | 1.29 (1.23, 1.34) | 1.59 (1.46, 1.73) | 1.29 (1.01, 1.66) | 1.51 (1.44, 1.59) |
| Convenience Stores | 1.21 (1.19, 1.22) | 1.16 (1.12, 1.20) | 1.36 (1.25, 1.48) | 1.13 (0.89, 1.45) | 1.30 (1.23, 1.36) |
| Bakeries, candy, ice cream | 1.36 (1.35, 1.38) | 1.36 (1.29, 1.42) | 1.44 (1.32, 1.56) | 0.82 (0.65, 1.03) | 1.48 (1.40, 1.55) |
| ^a^ Harmonized data for the 1990 census, 2000 census, and the 2008-2012 American Community Survey, published in the Longitudinal Tract Database product (LTDB), were used to estimate all percentage of each race/ethnicity within each tract. We defined a tract as predominantly one racial/ethnic group if greater than 60% of residents were any particular racial/ethnic group. Tracts that did not fall into any of these categories were classified as racially mixed areas.  ^b^ Food environment metrics derived from National Establishment Time Series (NETS) data 1990-2014 annually.  ^c^ Census tracts were excluded if they contained no land area (i.e. were water tracts) or had less than 50 residents. Urban tracts defined by RUCA=1.  ^d^ Estimates derived from generalized poisson regression models modeled as a function of time (in years), the census tract racial/ethnic composition, the population density, and the land area of the tract as an offset. | | | | | |
| **Table S7: Racial/ethnic disparities^b^ in food environment measures^a^ over time (1990-2014) for non-water US census tracts included in urban (defined by RUCA=1 or 2) sample^c^** | | | | | |
|  | **Predominantly non-Hispanic White** | **Predominantly non-Hispanic Black** | **Predominantly Hispanic** | **Predominantly Asian** | **Mixed Racial Composition** |
| **Disparities in 1990** | **Estimate (95% CI)^d^** | **Estimate (95% CI) ^d^** | **Estimate (95% CI) ^d^** | **Estimate (95% CI) ^d^** | **Estimate (95% CI) ^d^** |
| All Food Stores | Ref | 4.66 (3.91, 5.54) | 2.48 (1.39, 4.42) | 47.23 (26.58, 83.93) | 1.58 (1.32, 1.89) |
| Healthy Food Sales | Ref | 3.52 (2.92, 4.23) | 2.05 (1.14, 3.69) | 46.92 (26.57, 82.86) | 1.28 (1.07, 1.54) |
| All Supermarkets | Ref | 3.43 (2.85, 4.13) | 1.89 (1.05, 3.39) | 32.66 (16.59, 64.28) | 1.28 (1.07, 1.54) |
| Fruit and Vegetable Markets | Ref | 3.98 (3.20, 4.95) | 3.28 (1.79, 6.00) | 117.07 (59.10, 231.92) | 1.35 (1.11, 1.65) |
| All Unhealthy Food Sources | Ref | 4.36 (3.66, 5.20) | 2.25 (1.26, 4.01) | 35.17 (19.42, 63.71) | 1.66 (1.39, 1.99) |
| Fast Food | Ref | 3.23 (2.68, 3.89) | 1.60 (0.89, 2.87) | 15.58 (8.42, 28.83) | 1.59 (1.32, 1.91) |
| Convenience Stores | Ref | 4.25 (3.57, 5.07) | 1.74 (0.98, 3.12) | 8.14 (4.27, 15.53) | 1.52 (1.27, 1.82) |
| Bakeries, candy, ice cream | Ref | 1.97 (1.63, 2.39) | 2.12 (1.18, 3.79) | 60.65 (31.95, 115.16) | 1.40 (1.16, 1.68) |
| **Disparities in 2014** |  |  |  |  |  |
| All Food Stores | Ref | 5.39 (4.53, 6.41) | 3.64 (2.29, 5.79) | 22.01 (16.41, 29.54) | 3.10 (2.70, 3.55) |
| Healthy Food Sales | Ref | 3.86 (3.21, 4.63) | 3.47 (2.17, 5.53) | 17.78 (12.36, 25.57) | 2.71 (2.36, 3.12) |
| All Supermarkets | Ref | 3.70 (3.08, 4.46) | 2.26 (1.42, 3.60) | 12.54 (8.33, 18.87) | 2.57 (2.23, 2.96) |
| Fruit and Vegetable Markets | Ref | 4.32 (3.51, 5.31) | 7.29 (4.53, 11.75) | 32.98 (21.78, 49.95) | 3.17 (2.69, 3.72) |
| All Unhealthy Food Sources | Ref | 4.56 (3.83, 5.43) | 2.94 (1.85, 4.67) | 17.88 (13.67, 23.39) | 2.96 (2.58, 3.40) |
| Fast Food | Ref | 3.74 (3.13, 4.47) | 2.41 (1.51, 3.84) | 13.84 (10.45, 18.33) | 2.88 (2.51, 3.32) |
| Convenience Stores | Ref | 5.76 (4.85, 6.85) | 2.76 (1.73, 4.38) | 8.08 (5.91, 11.04) | 2.81 (2.45, 3.22) |
| Bakeries, candy, ice cream | Ref | 2.91 (2.43, 3.48) | 2.84 (1.79, 4.52) | 20.59 (15.27, 27.78) | 2.68 (2.33, 3.09) |
| **Time Trend 1990-2014** |  |  |  |  |  |
| All Food Stores | 1.17 (1.16, 1.18) | 1.24 (1.17, 1.33) | 1.38 (1.14, 1.66) | 0.85 (0.71, 1.02) | 1.55 (1.48, 1.62) |
| Healthy Food Sales | 1.03 (1.02, 1.04) | 1.07 (1.00, 1.15) | 1.28 (1.06, 1.55) | 0.69 (0.56, 0.85) | 1.41 (1.34, 1.48) |
| All Supermarkets | 0.98 (0.97, 0.99) | 1.01 (0.94, 1.09) | 1.06 (0.87, 1.28) | 0.66 (0.49, 0.89) | 1.31 (1.25, 1.38) |
| Fruit and Vegetable Markets | 1.24 (1.21, 1.26) | 1.28 (1.17, 1.40) | 1.73 (1.41, 2.12) | 0.73 (0.56, 0.95) | 1.76 (1.64, 1.90) |
| All Unhealthy Food Sources | 1.37 (1.36, 1.38) | 1.40 (1.31, 1.49) | 1.53 (1.27, 1.85) | 1.03 (0.85, 1.26) | 1.74 (1.66, 1.83) |
| Fast Food | 1.40 (1.39, 1.42) | 1.49 (1.39, 1.60) | 1.66 (1.37, 2.02) | 1.34 (1.04, 1.71) | 1.80 (1.71, 1.89) |
| Convenience Stores | 1.18 (1.16, 1.19) | 1.33 (1.25, 1.42) | 1.42 (1.18, 1.72) | 1.17 (0.92, 1.49) | 1.52 (1.45, 1.59) |
| Bakeries, candy, ice cream | 1.34 (1.32, 1.35) | 1.57 (1.46, 1.69) | 1.51 (1.25, 1.83) | 0.85 (0.68, 1.07) | 1.76 (1.67, 1.85) |
| ^a^ Harmonized data for the 1990 census, 2000 census, and the 2008-2012 American Community Survey, published in the Longitudinal Tract Database product (LTDB), were used to estimate all percentage of each race/ethnicity within each tract. We defined a tract as predominantly one racial/ethnic group if greater than 60% of residents were any particular racial/ethnic group. Tracts that did not fall into any of these categories were classified as racially mixed areas.  ^b^ Food environment metrics derived from National Establishment Time Series (NETS) data 1990-2014 annually.  ^c^ Census tracts were excluded if they contained no land area (i.e. were water tracts) or had less than 50 residents. Urban tracts defined by RUCA=1 or 2  ^d^ Estimates derived from generalized poisson regression models modeled as a function of time (in years), the census tract racial/ethnic composition, the population density, and the land area of the tract as an offset. | | | | | |

| **Table S8: Income disparities^b^ in food environment measures^a^ over time (1990-2014) for non-water US census tracts included in urban (defined by MSA) sample^c^** | | | | |
| --- | --- | --- | --- | --- |
|  | **Low Income** | **Middle Income** | **High Income** |  |
| **Disparities in 1990** | **Estimate (95% CI) ^d^** | **Estimate (95% CI) ^d^** | **Estimate (95% CI)^d^** |  |
| All Food Stores | 0.56 (0.49, 0.64) | 0.44 (0.40, 0.50) | Ref |  |
| Healthy Food Sales | 0.47 (0.41, 0.54) | 0.44 (0.39, 0.49) | Ref |  |
| All Supermarkets | 0.48 (0.42, 0.55) | 0.44 (0.39, 0.50) | Ref |  |
| Fruit and Vegetable Markets | 0.46 (0.39, 0.53) | 0.41 (0.36, 0.47) | Ref |  |
| All Unhealthy Food Sources | 0.57 (0.50, 0.66) | 0.45 (0.40, 0.50) | Ref |  |
| Fast Food | 0.52 (0.45, 0.60) | 0.45 (0.40, 0.51) | Ref |  |
| Convenience Stores | 0.65 (0.56, 0.74) | 0.54 (0.48, 0.61) | Ref |  |
| Bakeries, candy, ice cream | 0.39 (0.34, 0.45) | 0.35 (0.31, 0.40) | Ref |  |
| **Disparities in 2014** |  |  |  |  |
| All Food Stores | 1.02 (0.87, 1.18) | 0.60 (0.54, 0.67) | Ref |  |
| Healthy Food Sales | 0.94 (0.81, 1.11) | 0.60 (0.53, 0.67) | Ref |  |
| All Supermarkets | 0.87 (0.74, 1.02) | 0.59 (0.52, 0.66) | Ref |  |
| Fruit and Vegetable Markets | 1.18 (0.99, 1.40) | 0.64 (0.56, 0.72) | Ref |  |
| All Unhealthy Food Sources | 0.98 (0.84, 1.14) | 0.60 (0.54, 0.67) | Ref |  |
| Fast Food | 0.95 (0.81, 1.11) | 0.63 (0.56, 0.70) | Ref |  |
| Convenience Stores | 1.39 (1.19, 1.62) | 0.80 (0.72, 0.89) | Ref |  |
| Bakeries, candy, ice cream | 0.67 (0.57, 0.78) | 0.50 (0.44, 0.55) | Ref |  |
| **Time Trend 1990-2014** |  |  |  |  |
| All Food Stores | 1.42 (1.37, 1.48) | 1.26 (1.22, 1.30) | 1.11 (1.08, 1.15) |  |
| Healthy Food Sales | 1.28 (1.22, 1.33) | 1.09 (1.06, 1.13) | 0.96 (0.93, 0.99) |  |
| All Supermarkets | 1.17 (1.12, 1.22) | 1.03 (0.99, 1.06) | 0.91 (0.88, 0.95) |  |
| Fruit and Vegetable Markets | 1.70 (1.62, 1.79) | 1.38 (1.32, 1.44) | 1.14 (1.10, 1.19) |  |
| All Unhealthy Food Sources | 1.62 (1.56, 1.68) | 1.46 (1.41, 1.51) | 1.30 (1.25, 1.34) |  |
| Fast Food | 1.69 (1.62, 1.76) | 1.51 (1.46, 1.56) | 1.32 (1.27, 1.36) |  |
| Convenience Stores | 1.47 (1.41, 1.53) | 1.26 (1.22, 1.30) | 1.07 (1.04, 1.11) |  |
| Bakeries, candy, ice cream | 1.58 (1.52, 1.65) | 1.45 (1.40, 1.50) | 1.26 (1.22, 1.30) |  |
| ^a^ Harmonized data for the 1990 census, 2000 census, and the 2008-2012 American Community Survey, published in the Longitudinal Tract Database product (LTDB), were used to estimate inflation-adjusted, median household income within each tract. Categories represent tertiles.  ^b^ Food environment metrics derived from National Establishment Time Series (NETS) data 1990-2014 annually.  ^c^ Census tracts were excluded if they contained no land area (i.e. were water tracts) or had less than 50 residents. Urban tracts defined by being in MSA  ^d^ Estimates derived from generalized poisson regression models modeled as a function of time (in years), the census tract income composition, the population density, and the land area of the tract as an offset. | | | | |

| **Table S9: Income disparities^b^ in food environment measures^a^ over time (1990-2014) for non-water US census tracts included in urban (defined by RUCA=1) sample^c^** | | | | |
| --- | --- | --- | --- | --- |
|  | **Low Income** | **Middle Income** | **High Income** |  |
| **Disparities in 1990** | **Estimate (95% CI) ^d^** | **Estimate (95% CI) ^d^** | **Estimate (95% CI)^d^** |  |
| All Food Stores | 2.42 (2.17, 2.69) | 1.18 (1.06, 1.31) | Ref |  |
| Healthy Food Sales | 2.01 (1.80, 2.25) | 1.16 (1.04, 1.29) | Ref |  |
| All Supermarkets | 2.00 (1.79, 2.24) | 1.17 (1.05, 1.31) | Ref |  |
| Fruit and Vegetable Markets | 2.17 (1.89, 2.49) | 1.12 (0.98, 1.27) | Ref |  |
| All Unhealthy Food Sources | 2.52 (2.26, 2.81) | 1.23 (1.11, 1.37) | Ref |  |
| Fast Food | 2.36 (2.11, 2.64) | 1.31 (1.17, 1.46) | Ref |  |
| Convenience Stores | 2.72 (2.44, 3.03) | 1.40 (1.26, 1.56) | Ref |  |
| Bakeries, candy, ice cream | 1.74 (1.55, 1.95) | 1.00 (0.90, 1.12) | Ref |  |
| **Disparities in 2014** |  |  |  |  |
| All Food Stores | 2.83 (2.60, 3.09) | 1.24 (1.12, 1.39) | Ref |  |
| Healthy Food Sales | 2.55 (2.32, 2.80) | 1.23 (1.10, 1.38) | Ref |  |
| All Supermarkets | 2.27 (2.07, 2.49) | 1.21 (1.08, 1.36) | Ref |  |
| Fruit and Vegetable Markets | 3.48 (3.07, 3.93) | 1.29 (1.14, 1.47) | Ref |  |
| All Unhealthy Food Sources | 2.71 (2.49, 2.96) | 1.24 (1.12, 1.39) | Ref |  |
| Fast Food | 2.63 (2.41, 2.88) | 1.33 (1.19, 1.49) | Ref |  |
| Convenience Stores | 3.86 (3.54, 4.20) | 1.61 (1.45, 1.80) | Ref |  |
| Bakeries, candy, ice cream | 1.88 (1.72, 2.05) | 1.04 (0.94, 1.17) | Ref |  |
| **Time Trend 1990-2014** |  |  |  |  |
| All Food Stores | 1.36 (1.31, 1.41) | 1.30 (1.26, 1.35) | 1.27 (1.24, 1.30) |  |
| Healthy Food Sales | 1.21 (1.16, 1.26) | 1.12 (1.08, 1.16) | 1.09 (1.07, 1.12) |  |
| All Supermarkets | 1.10 (1.06, 1.15) | 1.06 (1.02, 1.10) | 1.05 (1.02, 1.07) |  |
| Fruit and Vegetable Markets | 1.59 (1.51, 1.68) | 1.39 (1.33, 1.46) | 1.31 (1.26, 1.36) |  |
| All Unhealthy Food Sources | 1.52 (1.47, 1.58) | 1.48 (1.43, 1.54) | 1.48 (1.44, 1.51) |  |
| Fast Food | 1.56 (1.50, 1.62) | 1.50 (1.45, 1.56) | 1.49 (1.46, 1.53) |  |
| Convenience Stores | 1.40 (1.35, 1.46) | 1.29 (1.24, 1.33) | 1.21 (1.18, 1.24) |  |
| Bakeries, candy, ice cream | 1.47 (1.42, 1.53) | 1.45 (1.40, 1.51) | 1.43 (1.39, 1.46) |  |
| ^a^ Harmonized data for the 1990 census, 2000 census, and the 2008-2012 American Community Survey, published in the Longitudinal Tract Database product (LTDB), were used to estimate inflation-adjusted, median household income within each tract. Categories represent tertiles.  ^b^ Food environment metrics derived from National Establishment Time Series (NETS) data 1990-2014 annually.  ^c^ Census tracts were excluded if they contained no land area (i.e. were water tracts) or had less than 50 residents. Urban defined by RUCA=1  ^d^ Estimates derived from generalized poisson regression models modeled as a function of time (in years), the census tract income composition, the population density, and the land area of the tract as an offset. | | | | |

| **Table S10: Income disparities^b^ in food environment measures^a^ over time (1990-2014) for non-water US census tracts included in urban (defined by RUCA=1 or 2) sample^c^** | | | | |
| --- | --- | --- | --- | --- |
|  | **Low Income** | **Middle Income** | **High Income** |  |
| **Disparities in 1990** | **Estimate (95% CI) ^d^** | **Estimate (95% CI) ^d^** | **Estimate (95% CI)^d^** |  |
| All Food Stores | 0.60 (0.53, 0.68) | 0.51 (0.46, 0.57) | Ref |  |
| Healthy Food Sales | 0.49 (0.43, 0.56) | 0.50 (0.45, 0.55) | Ref |  |
| All Supermarkets | 0.49 (0.43, 0.56) | 0.50 (0.45, 0.56) | Ref |  |
| Fruit and Vegetable Markets | 0.49 (0.43, 0.57) | 0.47 (0.42, 0.54) | Ref |  |
| All Unhealthy Food Sources | 0.61 (0.54, 0.70) | 0.52 (0.47, 0.58) | Ref |  |
| Fast Food | 0.55 (0.49, 0.63) | 0.53 (0.47, 0.59) | Ref |  |
| Convenience Stores | 0.68 (0.60, 0.78) | 0.62 (0.56, 0.69) | Ref |  |
| Bakeries, candy, ice cream | 0.41 (0.36, 0.47) | 0.41 (0.37, 0.46) | Ref |  |
| **Disparities in 2014** |  |  |  |  |
| All Food Stores | 1.26 (1.10, 1.45) | 0.67 (0.60, 0.74) | Ref |  |
| Healthy Food Sales | 1.17 (1.02, 1.34) | 0.66 (0.59, 0.73) | Ref |  |
| All Supermarkets | 1.06 (0.92, 1.22) | 0.64 (0.57, 0.72) | Ref |  |
| Fruit and Vegetable Markets | 1.50 (1.28, 1.75) | 0.70 (0.62, 0.79) | Ref |  |
| All Unhealthy Food Sources | 1.21 (1.06, 1.38) | 0.66 (0.60, 0.74) | Ref |  |
| Fast Food | 1.17 (1.02, 1.34) | 0.69 (0.62, 0.77) | Ref |  |
| Convenience Stores | 1.72 (1.50, 1.97) | 0.88 (0.79, 0.99) | Ref |  |
| Bakeries, candy, ice cream | 0.83 (0.73, 0.95) | 0.55 (0.49, 0.61) | Ref |  |
| **Time Trend 1990-2014** |  |  |  |  |
| All Food Stores | 1.52 (1.48, 1.57) | 1.24 (1.20, 1.28) | 1.11 (1.08, 1.15) |  |
| Healthy Food Sales | 1.37 (1.33, 1.42) | 1.08 (1.04, 1.11) | 0.96 (0.93, 0.99) |  |
| All Supermarkets | 1.25 (1.21, 1.29) | 1.01 (0.98, 1.04) | 0.91 (0.88, 0.94) |  |
| Fruit and Vegetable Markets | 1.82 (1.74, 1.90) | 1.35 (1.29, 1.41) | 1.15 (1.10, 1.19) |  |
| All Unhealthy Food Sources | 1.72 (1.67, 1.77) | 1.43 (1.39, 1.48) | 1.30 (1.26, 1.34) |  |
| Fast Food | 1.80 (1.74, 1.86) | 1.48 (1.43, 1.52) | 1.32 (1.27, 1.36) |  |
| Convenience Stores | 1.57 (1.52, 1.62) | 1.24 (1.20, 1.27) | 1.07 (1.04, 1.10) |  |
| Bakeries, candy, ice cream | 1.69 (1.63, 1.74) | 1.42 (1.38, 1.47) | 1.26 (1.22, 1.30) |  |
| ^a^ Harmonized data for the 1990 census, 2000 census, and the 2008-2012 American Community Survey, published in the Longitudinal Tract Database product (LTDB), were used to estimate inflation-adjusted, median household income within each tract. Categories represent tertiles.  ^b^ Food environment metrics derived from National Establishment Time Series (NETS) data 1990-2014 annually.  ^c^ Census tracts were excluded if they contained no land area (i.e. were water tracts) or had less than 50 residents. Urban tracts defined by RUCE=1 or 2  ^d^ Estimates derived from generalized poisson regression models modeled as a function of time (in years), the census tract income, the population density, and the land area of the tract as an offset. | | | | |
